# L1CAM from human melanoma carries a novel type of N-glycan with Galβ1-4Galβ1- motif. Involvement of N-linked glycans in migratory and invasive behaviour of melanoma cells

**DOI:** 10.1007/s10719-012-9374-5

**Published:** 2012-04-29

**Authors:** Dorota Hoja-Łukowicz, Paweł Link-Lenczowski, Andrea Carpentieri, Angela Amoresano, Ewa Pocheć, Konstantin A. Artemenko, Jonas Bergquist, Anna Lityńska

**Affiliations:** 1 Institute of Zoology, Jagiellonian University, 9 Gronostajowa Street, 30-387 Krakow, Poland; 2 Department of Organic Chemistry and Biochemistry, School of Biotechnological Sciences, Federico II University of Naples, Naples, Italy; 3 Department of Chemistry - BMC, Analytical Chemistry, Uppsala University, P.O. Box 599, SE-751 24 Uppsala, Sweden; 4 Department of Chemistry – BMC, Analytical Chemistry and SciLifeLab, Uppsala University, P.O. Box 599, SE-751 24 Uppsala, Sweden

**Keywords:** L1CAM, Galβ1-4Galβ1-motif, *N*-glycolylneuraminic acid, Isogenic melanoma cells, NP HPLC, MALDI MS

## Abstract

Dramatic changes in glycan biosynthesis during oncogenic transformation result in the emergence of marker glycans on the cell surface. We analysed the *N-*linked glycans of L1CAM from different stages of melanoma progression, using high-performance liquid chromatography combined with exoglycosidase sequencing, matrix-assisted laser desorption/ionisation time-of-flight mass spectrometry, and lectin probes. L1CAM oligosaccharides are heavily sialylated, mainly digalactosylated, biantennary complex-type structures with galactose β1-4/3-linked to GlcNAc and with or without fucose α1-3/6-linked to GlcNAc. Hybrid, bisected hybrid, bisected triantennary and tetraantennary complex oligosaccharides, and β1-6-branched complex-type glycans with or without lactosamine extensions are expresses at lower abundance. We found that metastatic L1CAM possesses only α2-6-linked sialic acid and the loss of α2-3-linked sialic acid in L1CAM is a phenomenon observed during the transition of melanoma cells from VGP to a metastatic stage. Unexpectedly, we found a novel monoantennary complex-type oligosaccharide with a Galβ1-4Galβ1- epitope capped with sialic acid residues A1[3]G(4)2S_2-3_. To our knowledge this is the first report documenting the presence of this oligosaccharide in human cancer. The novel and unique *N-*glycan should be recognised as a new class of human melanoma marker. In functional tests we demonstrated that the presence of cell surface α2-3-linked sialic acid facilitates the migratory behaviour and increases the invasiveness of primary melanoma cells, and it enhances the motility of metastatic cells. The presence of cell surface α2-6-linked sialic acid enhances the invasive potential of both primary and metastatic melanoma cells. Complex-type oligosaccharides in L1CAM enhance the invasiveness of metastatic melanoma cells.

**Electronic supplementary material:**

The online version of this article (doi:10.1007/s10719-012-9374-5) contains supplementary material, which is available to authorized users.

## Introduction

Alterations of the cell surface carbohydrate structures of cancer cells are suggested to affect normal cellular interactions and have been shown to facilitate tumour cell colonisation and metastasis. The cancer-associated changes include underexpression and overexpression of naturally occurring glycans, the appearance of incomplete or truncated structures, the appearance of novel structures, and neoexpression of glycans normally restricted to embryonic tissues [1–3]. One such frequently observed membrane modification is altered expression of β1-6-branched *N-*linked glycans, caused by increased transcription and activity of β1,6-*N-*acetylglucosaminyltransferase V (GnT-V). The β1-6-branched *N-*linked oligosaccharides are believed to aid metastasis by making the cells more invasive, although the mechanism is not very clear [4, 5]. Most of the cell lines expressing these oligosaccharides have been shown to metastasize to either the liver or the lungs [6, 7]. β1-6 branching facilitates the synthesis of poly-*N-*acetyllactosamine repeats and Lewis structures, as well as terminal capping by sialic acid [1]. The *N-*acetyllactosamine unit and poly-*N-*acetyllactosamine repeats should serve as high-affinity ligands for galectins, which are expressed in various cells and tissues including the lung endothelium [1, 8]. The sialylated Lewis antigens on these oligosaccharides are involved in selectin-mediated adhesion of cancer cells to the vascular endothelium, and these determinants are thought to be closely associated with hematogenous metastasis of cancer [2]. A positive correlation has been demonstrated between the metastatic ability of various cell lines and increased cell surface sialylation and/or differences in the position of sialic residues [1, 9]. To understand the role of these oligosaccharides in tumour progression it is important to determine the terminal substitutions and identify the glycoproteins that carry them. In malignant transformation the abnormal oligosaccharides are widely expressed on cell adhesion molecules (CAMs) such as β1 and β3 integrin subunits, a few of the α integrin subunits, CD44, and cadherins [10–15]. Another one of these membrane-bound CAMs, known to be a valuable marker for poor prognosis in several types of cancer, is the L1cell adhesion molecule.

The L1cell adhesion molecule (L1CAM, CD171), a member of the immunoglobulin (Ig) superfamily, is a 200–220 kDa transmembrane glycoprotein. The ectodomain, consisting of six immunoglobulin-like domains and five fibronectin-like repeats (type III), is linked *via* a single transmembrane sequence to a phylogenetically conserved cytoplasmic domain. L1CAM possesses 21 potential *N-*glycosylation sites located on Ig domains and FNIII domains of the ectodomain (http://www.ncbi.nlm.nih.gov/PROW/guide/1813332452_g.htm), but the pattern of glycosylation is still unknown. L1CAM was initially identified in neural cells, but its expression has also been seen in some other normal tissues and in several types of human carcinomas and melanomas [16–20]. L1CAM expression in carcinomas increases the dissemination of tumour cells by enabling cell migration and invasion [16, 18] and promotes the epithelial–mesenchymal transition [19]. In melanoma the expression of L1CAM occurs in a stage-dependent manner; L1CAM is expressed in primary melanomas and cutaneous metastases and not in melanocytic nevi and melanocytes [18].

The mechanism by which the L1 cell adhesion molecule contributes to tumour progression has not been clearly established. In this work we used HPLC and mass spectrometry techniques to determine the *N-*glycan profile of the L1 cell adhesion molecule from primary vertical growth phase (VGP) and metastatic melanoma cells. Unexpectedly, we found an unknown monoantennary complex-type structure with a novel oligosaccharide branch on the C3-linked antenna. Using lectins, molecules which recognise and bind specific mono- and oligosaccharides, we confirmed the findings from structural studies. In functional tests we showed the importance of L1CAM glycans in the migratory and invasive behaviour of melanoma cells.

## Materials and methods

### Materials

Unsubstituted and substituted lectins, FITC-Avidin as well as Vectashield Hardset™ mounting medium with DAPI were purchased from Vector (USA). Anti-mouse IgG/AP, and DIG Glycan Differentiation Kit were purchased from Boehringer (Mannheim, Germany). Immunoprecipitation Kit (Protein G), *N-*glycosidase F (PNGase F), and neuraminidase from *Arthrobacter ureafaciens* were purchased from Roche (Mannheim, Germany). Sialidase A, β-galactosidase (*Streptococcus pneumoniae*), β-*N-*acetylhexosaminidase (Jack Bean), α-fucosidase (Bovine Kidney), α-mannosidase (Jack Bean), Signal 2-AB Labelling Kit as well as GlycoSep N column were from Prozyme (San Leandro, CA). Immobilon-P transfer membrane, Micropure-EZ column and rabbit anti-mouse IgG/AP were obtained from Millipore and Chemicon (USA), respectively. Mouse anti-human L1CAM mAbs, clone UJ127.11, α2-3-neuraminidase from *Streptococcus pneumonia,* RPMI 1640 with GlutaMax-I medium (Gibco, UK), Protein Assay Kit, ExtrAvidin/AP, normal goat serum, protease inhibitor cocktail, swainsonine, GlycoProfile 2-AB Labelling Kit and GlycoProfile Glycan Clean-up columns were obtained from Sigma (St. Louis, MO). Alexa Fluor Cy3-conjugated goat anti-mouse mAbs, and foetal bovine serum were from Invitrogen (USA). Carbograph SPE Extract-Clean columns were obtained from Alltech (Deerfield, IL). BD Falcon FluoroBlok™ 96-Multiwell Insert System, 96-well BD BioCoat™ Tumor Invasion System and DiIC12(3) fluorescent dye were from BD Biosciences. All other chemicals were of the highest purity and were purchased from Sigma (St. Louis, MO).

### Cell lines

Human cutaneous primary melanoma cell lines: WM793 (VGP), FM-55-P, IGR-39, WM1552C (RGP), WM75 (VGP) and metastatic melanoma cell lines: WM1205Lu (lung metastasis), M6/B7 and UKRV-Mel-15a were obtained from the ESTDAB Melanoma Cell Bank (Tübingen). Metastatic melanoma cell line Ma-Mel-27 was kindly donated by Prof. D. Schadendorf of the Klinik für Dermatologie, Venerologie und Allergologie (Universitätsklinikum Essen, Germany).

### Cell culture conditions and cell extract preparation

Cells were maintained in RPMI 1640 medium with GlutaMax-I supplemented with 10 % foetal bovine serum, 100 units/ml penicillin and 100 μg/ml streptomycin. Cells were grown in monolayers in a 5 % CO_2_ atmosphere at 37 °C in a humidified incubator. Cell extract proteins were prepared on ice by sonication (Bandelin Electronic) of cells in 50 mM Tris/HCl buffer (pH 7.5) containing 1 mM EDTA, protease inhibitor cocktail (20 μl/ml) and 1 mM PMSF. Triton X-100 (final concentration 1 %) and protamine sulfate (final concentration 0.3 %) were added and the homogenates were incubated for 30 min. Cell extracts were cleared by centrifugation at 18,000 × g for 20 min at 4 °C. The protein concentration was determined with the Protein Assay Kit. All cell cultures were free of *Mycoplasma* infection as verified by PCR and by DAPI staining under confocal microscopy.

### Isolation of L1CAM from cell extract

L1CAM was immunoprecipitated with a Protein G Immunoprecipitation Kit according to the manufacturer’s protocol. Briefly, cleared cell extracts of WM793 and WM1205Lu cell lines (10 mg total protein) were incubated with 50 μg UJ127.11 mAb for 18 h at 4 °C. Subsequently, 40 μl Protein G-agarose was added to each immunoprecipitate and incubated for another 4 h at 4 °C. Samples were boiled in 100 μl Laemmli sample buffer containing 5 % β-mercaptoethanol at 100 °C for 8 min. Both immunoprecipitates were electrophoresed in a 7.2 % SDS-polyacrylamide gel according to Laemmli [21] until the standard-mass protein of 116 kDa went out of the gel. After electrotransfer on a PVDF membrane, immunodetection of L1CAM was performed using mAbs UJ127.11 (1:12500 dilution) and rabbit anti-mouse IgG/AP (1:4000 dilution) as secondary antibody. The conjugated alkaline phosphatase was detected by NBT/X-phosphate staining.

### Release and purification of *N-*linked oligosaccharides

*N-*glycans were released from purified L1CAM by *in situ* digestion with PNGase F according to [22] with minor modifications. Briefly, individual protein bands corresponding to L1CAM were excised from the PVDF membrane, reduced, alkylated and treated with PNGase F to release *N-*linked glycans. Glycans were purified on Carbograph SPE Extract-Clean columns according to the manufacturer’s protocol and then dried *in vacuo.*

### Fluorescent labelling of the reducing terminus of oligosaccharides

Oligosaccharides were fluorescence-labelled with 2-AB by reductive amination using the GlycoProfile 2-AB Labelling Kit. Labelled oligosaccharides were purified on GlycoProfile Glycan Clean-up columns according to the manufacturer’s protocol and then dried *in vacuo.*

### Simultaneous oligosaccharide sequencing by exoglycosidase digestions

The 2-AB labelled oligosaccharides were split into six equal parts, five of which were incubated overnight at 37 °C in parallel with 3 μl of each of the following exoglycosidase arrays prepared in 20 mM sodium acetate buffer, pH 5.5: (i) *Arthrobacter ureafaciens* sialidase (α2-3,6,8 sialic acid, 2 U/ml); (ii) sialidase and *Streptococcus pneumoniae* β-galactosidase (β1-4 galactose, 80 mU/ml); (iii) sialidase, galactosidase, and bovine kidney α-fucosidase (α1-2,6,3,4 fucose, 1 U/ml); (iv) sialidase, galactosidase, fucosidase, and Jack bean β-*N-*acetylhexosaminidase (GlcNAc β1-2,3,4,6 > bisect, 30 U/ml); and (v) sialidase, galactosidase, fucosidase, *N*-acetylhexosaminidase, and Jack bean α-mannosidase (α1-2,6,3 mannose, 100 U/ml). Each reaction mixture was desalted and freed of enzymes by using Micropure-EZ column, according to manufacturer’s protocol. All samples were dried *in vacuo.*

### Normal-phase HPLC

2-AB-labelled sugars were separated on a 4.6 × 250 mm Glyco-Sep N column using two Shimadzu LC-10ADvp pumps and a Shimadzu RF-10Ax1 fluorescence detector (Japan). The gradient used was as described by [23]: solvent A, acetonitrile; solvent B, 50 mM ammonium formate, pH 4.4. Initial conditions were 20 % B at a flow rate of 0.4 ml/min followed by a linear gradient of 20–53 % B over 132 min followed by 53–100 % B over the next 3 min. The column was washed with 100 % B for 5 min at a flow rate of 1 ml/min before reequilibration in the initial solvent system.

### MALDI-TOF mass spectrometry

Positive Reflectron MALDI spectra were recorded on a Voyager DE STR instrument (Applied Biosystems, Framingham, MA). The MALDI matrix was prepared by dissolving 10 mg of 2,5-dihydroxybenzoic acid (2,5-DHB in acetonitrile/water (9:1 v/v). Typically, 1 μl of matrix was applied to the metallic sample plate and 1 μl of analyte was then added. Acceleration and reflector voltages were set up as follows: target voltage at 20 kV, first grid at 65 % of target voltage, delayed extraction at 400 ns to obtain the best signal-to-noise ratios and the best possible isotopic resolution with multipoint external calibration using calibrant mixture purchased from Applied Biosystems. Each spectrum represents the sum of 3,000 laser pulses from randomly chosen spots per sample position. Raw data were analysed using the computer software provided by the manufacturers and are reported as monoisotopic masses.

### Lectin precipitation and western blotting

The cleared cell extracts (250 μg total protein per sample) were diluted 1:1 (v/v) with incubation buffer containing 10 mM HEPES, 150 mM NaCl, 0.1 mM CaCl_2_ and 0.01 mM MgCl_2_, pH 7.5, and incubated overnight at 4 °C with 8 μl biotinylated lectins or with 40 μl agarose-bound lectins. Then the samples with biotinylated lectins were mixed with 40 μl streptavidin-agarose for another 4 h at 4 °C. All precipitates were washed three times with incubation buffer and once with PBS, with centrifugation each time (1,500 × g, 3 min). Precipitated glycoproteins were liberated by boiling in Laemmli sample buffer containing 5 % β-mercaptoethanol and 1 mM EDTA at 100 °C for 8 min. Electrophoresis and western blotting were performed as described above.

### Immunoprecipitation and lectin blotting

Cell extracts of WM793 and WM1205Lu cell lines (1.8 mg total protein each) were immunoprecipitated with 8 μg mAb UJ127.11 for 18 h at 4 °C. Next, 20 μl Protein G-agarose (Immunoprecipitation Kit) was added to each of the immunoprecipitates and incubated for another 4 h at 4 °C. The immunoprecipitates of the WM793 and WM1205Lu cell lines were divided into six equal parts for lectin probes (each part equivalent to 300 μg total protein). Electrophoresis, electrotransfer and immunodetection of L1CAM on PVDF membranes were performed as above. For *on blot* lectin probing we used the following digoxigenin-labelled GNA (1:2000 dilution; DIG Glycan Differentiation Kit) and biotin-labelled lectins (1:4000 dilution): MAA, SNA, LEA, PHA-E, PHA-L, DSA, AAA and UEA-I. After washing, the respective membranes were incubated with anti-digoxigenin-AP and ExtrAvidin-AP conjugate (1:4000 dilution) for 1 h at RT. Conjugated alkaline phosphatase was detected by NBT/X-phosphate staining.

### Double immunofluorescence

Cells were plated on glass slides and grown in four-well plates (Nunc, Germany) to reach 80 % confluence. Cells were fixed with 2 % PFA for 10 min at RT. After blocking with 10 % NGS and 2 % BSA/PBS for 30 min at RT the cells were incubated with biotinylated or FITC-conjugated lectin (dilution 1:500) in 1 % BSA/PBS for 2 h at RT. When biotinylated lectins were used an additional incubation was done with ExtraAvidin-AlexaFluor488 (dilution 1:100) in 1 % BSA/PBS for 2 h at RT. Then an incubation was performed with mAb UJ127.11 (diluted 1:100) in 1 % BSA/PBS overnight at RT, and then with Cy3-conjugated goat anti-mouse IgG (diluted 1:300) in 1 % BSA/PBS for 2 h at RT. Cells were mounted in Vectashield Hardset™ mounting medium with DAPI and observed under a confocal microscope (Zeiss LSM 510 META, Carl Zeiss MicroImaging GmbH, Jena, Germany).

### Wound healing assay

Scrape-wound healing assays were performed in a 6-well culture plate as described in detail by [11]. Briefly, WM793 and WM1205Lu cells were grown to confluence. After aspiration of the medium, the cell-coated surface was scraped with a 200 μl pipette tip in a single stripe. Then the surface was washed twice with RPMI 1640 and covered with medium supplemented with 10 % FBS. A photograph of each wound was taken through an inverted microscope with a digital camera (Canon Powershot G10). The wounds were allowed to heal for 22 h at 37 °C and then photographed. In some experiments the wound healing assay was performed in the presence of one of the following reagents: anti-L1CAM (UJ127.11; 10 μg/ml), swainsonine (SW; 10 μl/ml), MAA (50 μg/ml) or SNA (50 μg/ml). In other experiments we used one of the following reagent combinations: anti-L1CAM and SW, anti-L1CAM and MAA, or anti-L1CAM and SNA. The average extent of wound closure was quantified from twenty measurements of the width of the wound space in two separate trials for each of these treatments, using AxioVision software (Carl Zeiss). Values are expressed as the means ± standard deviation of three separate experiments. Changes in cell migration rate after antibody, lectin and SW treatments were calculated by comparing the migration of untreated (taken as 100 % migration) and treated cells.

### Matrigel invasion assay

Cells were grown in the presence or absence of SW (10 μg/ml) for 24 h at 37 °C in a 5 % CO_2_ atmosphere and then labelled with DiIC12(3) fluorescent dye. Invasion assays were performed using DiIC12(3) pre-labelled cells (12.5 × 10^3^ cells/well) suspended in serum-free RPMI 1640 and plated onto either uncoated (BD Falcon FluoroBlok™ 96-Multiwell Insert System) or matrigel-coated filters (96-well BD BioCoat™ Tumor Invasion System). As chemoattractant, 5 % FBS in RPMI 1640 medium was placed in the lower chambers. In the experimental treatments, tumour cells grown in the absence of SW before the invasion assays were plated in wells in the presence or absence of anti-L1CAM antibody (40 μg/ml; UJ127.11). Tumour cells grown in the presence of SW before invasion assays were plated in wells in the presence of SW (10 μg/ml) or in the presence of both the antibody (40 μg/ml; UJ127.11) and SW (10 μg/ml). In some experiments, after labelling with fluorescent dye and before invasion assays, tumour cells were desialylated with neuraminidases from *Arthrobacter ureafaciens* or *Streptococcus pneumoniae* for 1 h at 37 °C and then incubated in serum-free medium in the absence or presence of antibody (40 μg/ml; UJ127.11) for another 1 h at 37 °C. Finally, the tumour cells were seeded in the wells. After 17 h incubation at 37 °C in a 5 % CO_2_ atmosphere, the fluorescence of cells in the lower chambers was measured with a BioTek Synergy™ instrument. Each experiment was done two or three times, with four repetitions for each sample, and the mean values ± standard deviation were calculated.

## Results

The main aim of this study was to determine the *N-*glycosylation pattern of the L1CAM molecule at different stages of melanoma progression and to evaluate the effect of these glycans on melanoma cell behaviour. We used isogenic human melanoma cell lines: the weakly tumourigenic melanoma parental WM793 cell line, and its metastatic counterpart WM1205Lu from a spontaneous lung metastasis after subcutaneous injection of parental cells to mice. This approach eliminated the diversity of genetic backgrounds between individuals and eliminated variability due to sex, age, previous history, *etc.* Supplementary Figure S1 represents our research design. The advantage of our protocol is that it combined high performance liquid chromatography with sequential exoglycosidase digestion, revealing structural details such as anomericity and linkage positions, complemented by matrix-assisted laser desorption/ionisation time-of-flight (MALDI-TOF) mass spectrometry. We analysed the *N-*glycosylation pattern of L1CAM protein, corresponding to a molecular weight of 220 kDa. The whole pool of 2AB-labelled oligosaccharides was divided into six equal parts, five of which were subjected to exoglycosidase digestion arrays. Each pool was separately fractionated on a GlycoSep N column and analysed on the basis of the shift in the elution profile after treatment with the given exoglycosidase mixture and on the basis of changes in the relative areas of peaks, using the on-line GlycoBase 2 database. The elution position of each peak was described in glucose units (GU) by comparison with the elution positions of a standard 2AB-labelled dextran hydrolysate mixture [23]. In parallel, other portions of PNGase F-released and desialylated *N-*glycan pools were subjected to MALDI-TOF MS. Glycans were analysed on the basis of their pseudomolecular ion masses. The presence of the glycan structures found was confirmed after immunoprecipitation by *on blot* probing with a set of lectins, by lectin precipitation with subsequent western blotting, and by lectin staining in confocal microscopy. A scrape-wound assay and a matrigel invasion assay were done for the functional studies.

## Normal phase HPLC of L1CAM oligosaccharides from WM793 primary melanoma cells

**Fig. 1:**
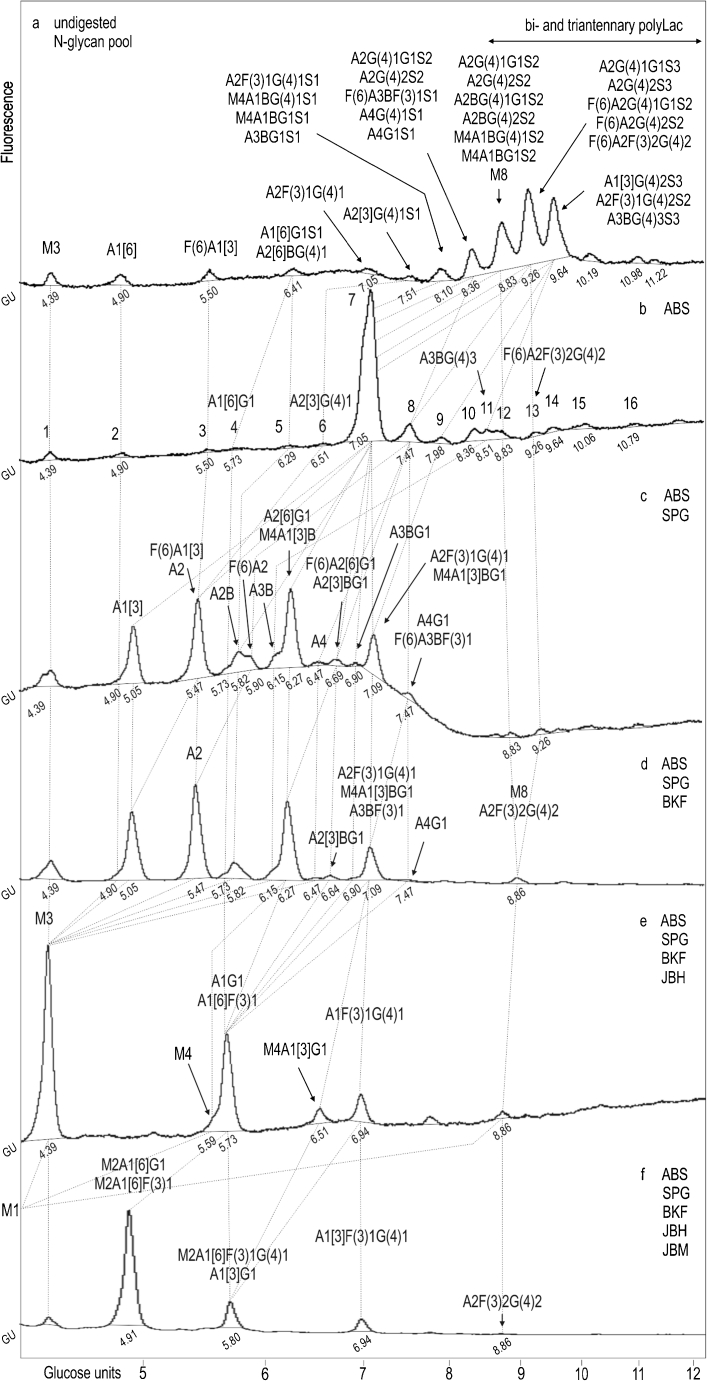
HPLC profiles of the *N*-glycan pool of L1CAM from primary WM793 cell line simultaneously digested with a series of enzyme arrays. HPLC analysis of the total glycan pool **a** and the products resulting from digestion of five aliquots of the total glycan pool with a series of enzyme arrays **b–f**. The enzymes used were as follows: ABS, *Arthrobacter ureafaciens* sialidase (removes all sialic acids); SPG, *Streptococcus pneumoniae* β-galactosidase (removes only β1-4-linked galactose); BKF, bovine kidney α-fucosidase (removes α1-2,6,3,4-linked fucose); JBH, jack bean α-*N*-acetylhexosaminidase (removes GlcNAc); JBM, jack bean α-mannosidase (removes α1-2,6,3-linked mannose). The glucose unit (GU) value of each peak was calculated by comparison with the dextran hydrolysate ladder shown at the bottom of the figure. Structures were assigned from the glucose unit values, the known incremental values for monosaccharide residues and the known specificity of the exoglycosidase enzymes. The structure abbreviations used were as follows: all *N-*glycans have two core GlcNAcs, Aa[3/6] indicates the number “a” of antennae on the trimannosyl core linked to the 3/6-mannose arm; G(4)b and Gc indicate the number “b” and “c” of terminal galactose residues β1-4- and β1-3-linked to antenna GlcNAc respectively; F(6) and F(3)d indicate a core fucose α1-6-linked to the core GlcNAc and “d” fucose residues α1-3-linked to antenna GlcNAc respectively; B, represents bisecting GlcNAc β1-4-linked to core mannose; Me represents the number “e” of mannose residues; Sf represents the number “f” of sialic acids linked to the galactose or *N-*acetylglucosamine in antennae. Dotted lines indicate the shifts of the glycans digested by the subsequent enzyme array. M1 structure with a GU value of 2.70 is outside the chromatogram (panel **f**)

**Fig. 2:**
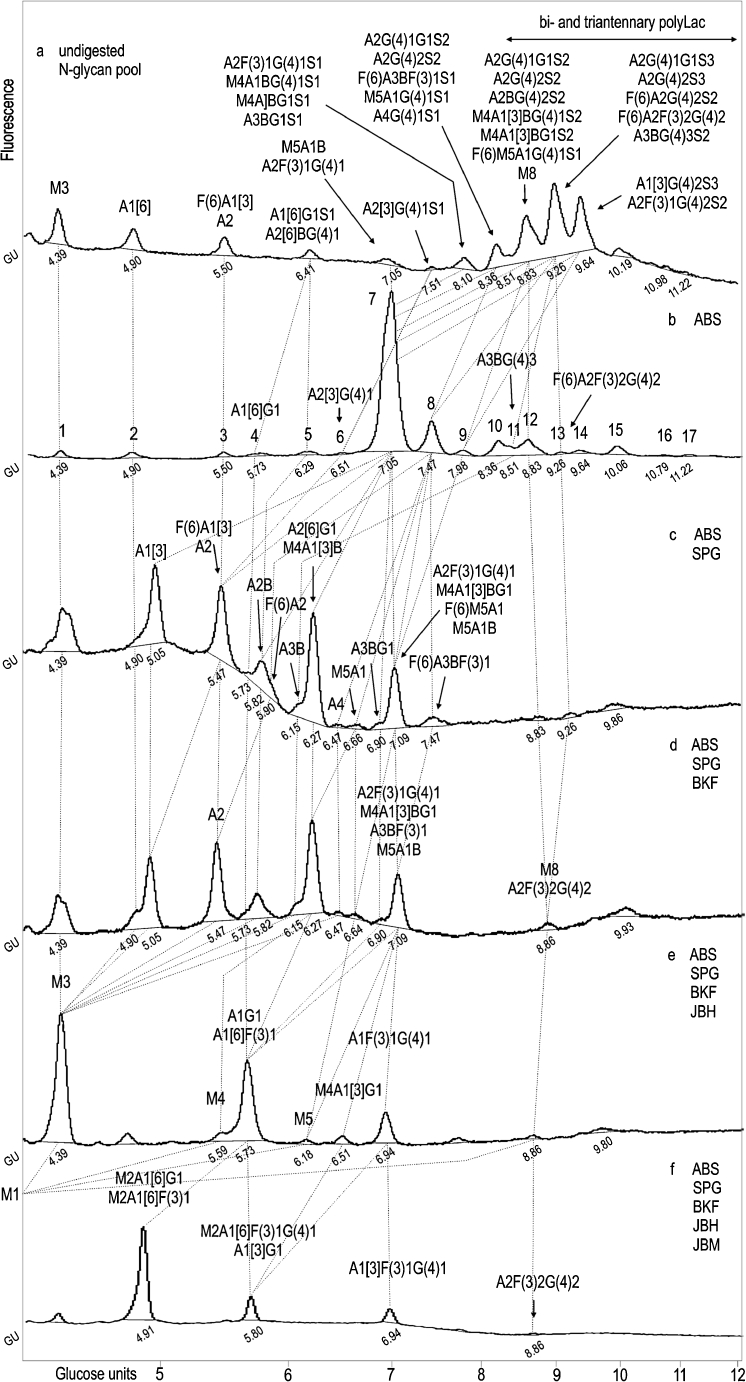
HPLC profiles of the *N*-glycan pool of L1CAM from metastatic WM1205Lu cell line simultaneously digested with a series of enzyme arrays. HPLC analysis of the total glycan pool **a** and the products resulting from the digestion of five aliquots of the total glycan pool with a series of enzyme arrays **b–f**. See Fig. 1 for legend and abbreviations

The 2AB-labelled untreated glycan pool was resolved on a GlycoSepN column into fourteen peaks with GU values from 4.39 to 11.22 (Fig. 1a). Treatment with *Arthrobacter ureafaciens* sialidase (ABS; specificity for α2-3,6,8-linked terminal NeuAc and NeuGc; Fig. 1b) resulted in the formation of a dominating broad peak with a GU value of 7.05 (peak 7) and at least sixteen other peaks. Upon digestion with a mixture of ABS and *Streptococcus pneumoniae* galactosidase (SPG; specificity for β1-4-linked galactose; Fig. 1c), the dominating broad peak 7 produced four higher peaks with GU values of 5.05, 5.47, 6.27 and 7.09. The peak with a GU value of 5.05 was not expected, because it had arisen from the peak with a GU value of 7.05 by losing 2.00 GU corresponding to two galactose residues on the C3-linked antenna [24]. This peak did not change its position after further treatment with a mixture of ABS, SPG and bovine kidney fucosidase (BKF; specificity for α1-2 or α1-6-linked fucose; Fig. 1d). Addition of Jack bean β-*N-*acetylhexosaminidase (JBH; specificity for β1-2,3,4,6-linked GlcNAc and GalNAc; Fig. 2e) resulted in the formation of M3 structure (GU 4.39) from this peak. We concluded that peak 7 (Fig. 1b) possessed A1 structure with a novel oligosaccharide branch on the C3-linked antenna (see legend to Fig. 1 for nomenclature) and we suggest as most probable a monoantennary complex-type structure: A1[3]G(4)2. The proposed structure was supported by the enzymatic specificity of SPG, which at a concentration below 100 mU/ml removes only galactose β1-4-linked to GlcNAc. Moreover, judging from this GU value and the presence of a neighbouring peak with a GU value of 4.90 corresponding to A1[6] structure, the considered structure should be branched on the C3-linked antenna. The peak with a GU value of 5.47 contained mainly A2 structure (Fig. 1c), which had arisen mainly from A2G(4)2 structure (peak 7; Fig. 1b) by losing two β1-4-linked Gal residues. This structure did not change its position after treatment with a mixture of ABS, SPG and BKF enzymes, but upon additional digestion with JBH enzyme it shifted to the peak with a GU value of 4.39 corresponding to M3 structure (Fig. 1e). A broad peak located between the two peaks with GU values of 5.47 and 6.27 (Fig. 1c) consisted, in fact, of three small peaks with GU values of 5.73, 5.82 and 5.90, which contained A1G1, A2B and F(6)A2 structures, respectively. The A2B and F(6)A2 structures had arisen mainly from A2BG(4)2 (peak 7) and F(6)A2G(4)2 (peak 8; Fig. 1b) by losing two β1-4-linked Gal residues, respectively. The A1[6]G1 structure originated from peak 4 (Fig. 1b). The A2[6]G1 structure was a major component of the peak with a GU value of 6.27 (Fig. 1c) and resulted from removal of one β1-4-linked Gal residue from digalactosylated biantennary complex oligosaccharide (peak 7; Fig. 1b). Upon digestion with ABS, SPG, BKF and JBH enzymes (Fig. 1e), this structure moved and made a substantial contribution to the peak with a GU value of 5.73. Addition of Jack bean mannosidase at a concentration of 100 U/ml (JBM; specificity for α1-2,3,6-linked mannose, Fig. 1f) resulted in the formation of a peak with a GU value of 4.91. JBM has broad specificity; although the enzyme will not cleave a single α1-6-linked mannose residue from core β-mannose, it will remove a single α1-3-linked mannose from core β-mannose [25]. In the considered structure, the Gal residue β1-3-linked to GlcNAc was located on the 6-arm of the core mannose residue as determined by the product of Jack bean mannosidase digestion. The peak with a GU value of 7.09 (Fig. 1c) contained mainly the monofucosylated and monogalactosylated biantennary glycan A2F(3)1G(4)1 that was present in peak 7 (Fig. 1b) as structure resistant to SPG treatment. Moreover, this structure had also arisen from the monofucosylated and digalactosylated biantennary glycan A2F(3)1G(4)2 (peak 9; Fig. 1b), which upon SPG digestion lost 0.87 GU, indicating the removal of one galactose. The presence of α1-3-linked fucose on outer-arm GlcNAc made it impossible to remove galactose using *S. pneumoaniae* galactosidase. Subsequent digestion with BKF exoglycosidase (Fig. 1d) did not change this GU value. Digestion with JBH enzyme caused the loss of one GlcNAc from the antenna and thereby the formation of A1F(3)1G(4)1 structure with a GU value of 6.94 (Fig. 1e). Further digestion with JBM showed the arm-specific location of fucose. One component was digested with the loss of 1.14 GU, indicating the removal of one α1-3-linked mannose residue; the second component was not digested (Fig. 1f). In a similar manner we identified hybrid, bisected hybrid, bisected triantennary, tetraantennary and digalactosylated arm α1-3-difucosylated biantennary complex oligosaccharides as minor constituents of the *N-*glycan pool.

## Normal phase HPLC of L1CAM oligosaccharides from WM1205Lu metastatic melanoma cells

HPLC data for the 2AB-labelled glycan pool of L1CAM from metastatic melanoma cells (Fig. 2) were analysed as was done for the primary analogue. Briefly, HPLC analyses of *N-*glycans from primary and metastatic L1CAM showed qualitative and quantitative similarities (Table 1). L1CAM oligosaccharides were heavily sialylated (83.4 % for primary and 75.0 % for metastatic L1CAM). They were mainly digalactosylated, biantennary complex-type structures (48.8 % for primary and 41.4 % for metastatic L1CAM). The new monoantennary structure with a Galβ1-4Galβ1- motif comprised 15.9 % of primary and 16.4 % of metastatic L1CAM oligosaccharides. Multiantennary complex-type oligosaccharides were present at a low level in both glycan pools (5.4 % for primary and 5 % for metastatic L1CAM). The amount of bisected glycans was as much as three times higher. There were slightly more bisected structures in the primary (19.2 %) than in the metastatic (15.0 %) L1CAM molecule. One fourth of all structures possessed Galβ1-3-linked to GlcNAc residue (type 1 LacNAc). Interestingly, for digalactosylated, biantennary complex-type oligosaccharides the type 1 LacNAc:type 2 LacNAc ratio was reversed to a higher value in the metastatic glycan pool. This observation is in accord with well-documented changes in the ratio of type 1:type 2 chains during carcinogenesis [26]. Furthermore, β1-3-linked Gal residues were present almost exclusively on the 6-arm in complex-type glycans, suggesting the presence of site-specific β3-galactosyltransferase in melanoma. L1CAM from primary melanoma cells possessed 5.5 % core fucosylated structures and 5.5 % Le^x^ structures. In metastatic L1CAM the level of core fucosylated species was lower at 3.6 %, but the amount of Le^x^ antigen was higher at 6.9 %. Unlike type 2 LacNAc, type 1 LacNAc was not substituted by fucose on the GlcNAc residue, which prevented the appearance of Le^a^ structures. Left unidentified were the structures of 10.7 % of primary and 14.7 % of metastatic L1CAM. They might include bi- and triantennary polylactosamine complex-type oligosaccharides. The undigested sialylated forms of all structures found are proposed on the basis of the elution positions and the relative areas of the peaks after fractionation of undigested and ABS-treated glycan pools. The carbohydrate structures deduced from this work are shown in Table 1.

**Table 1:**
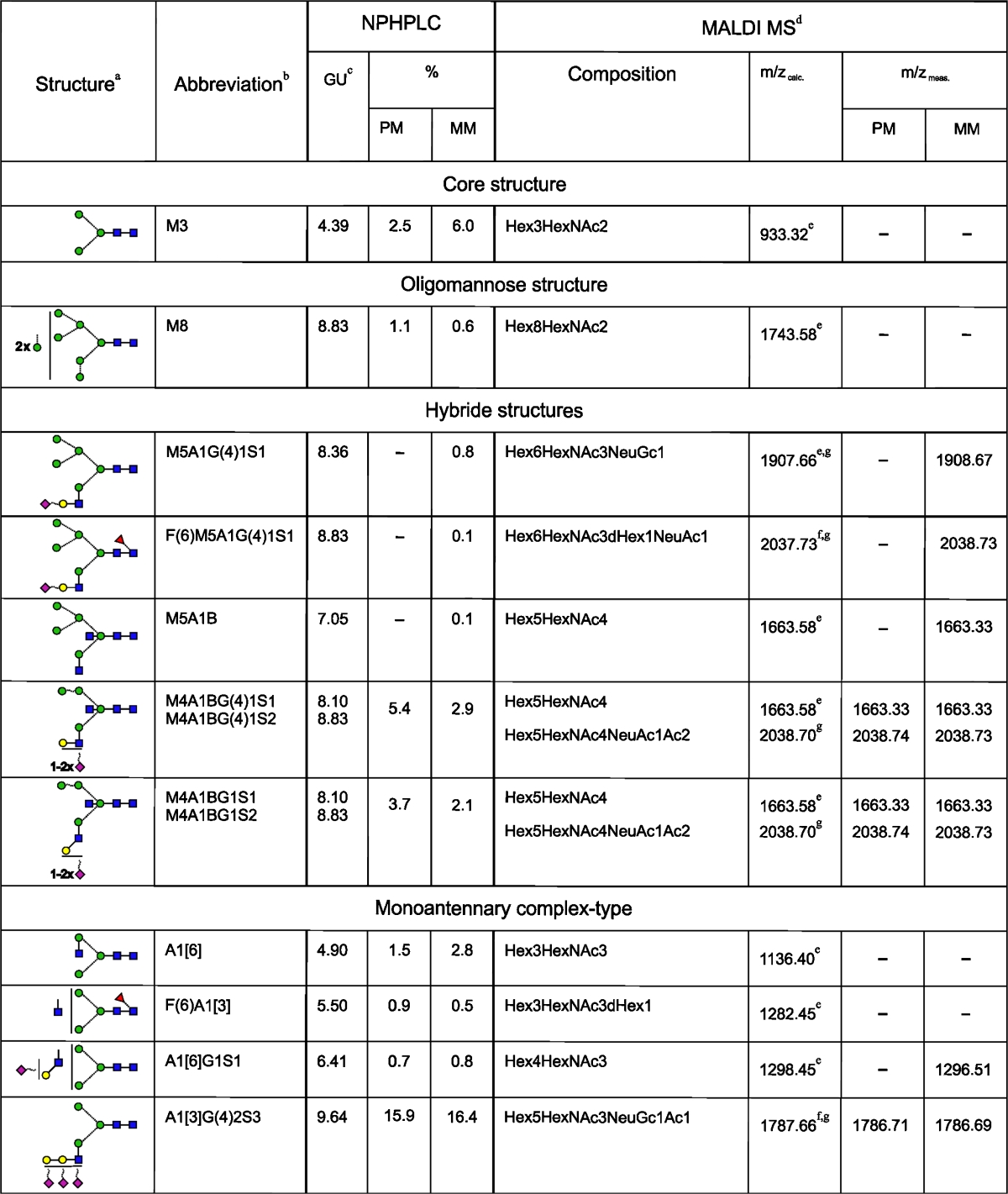

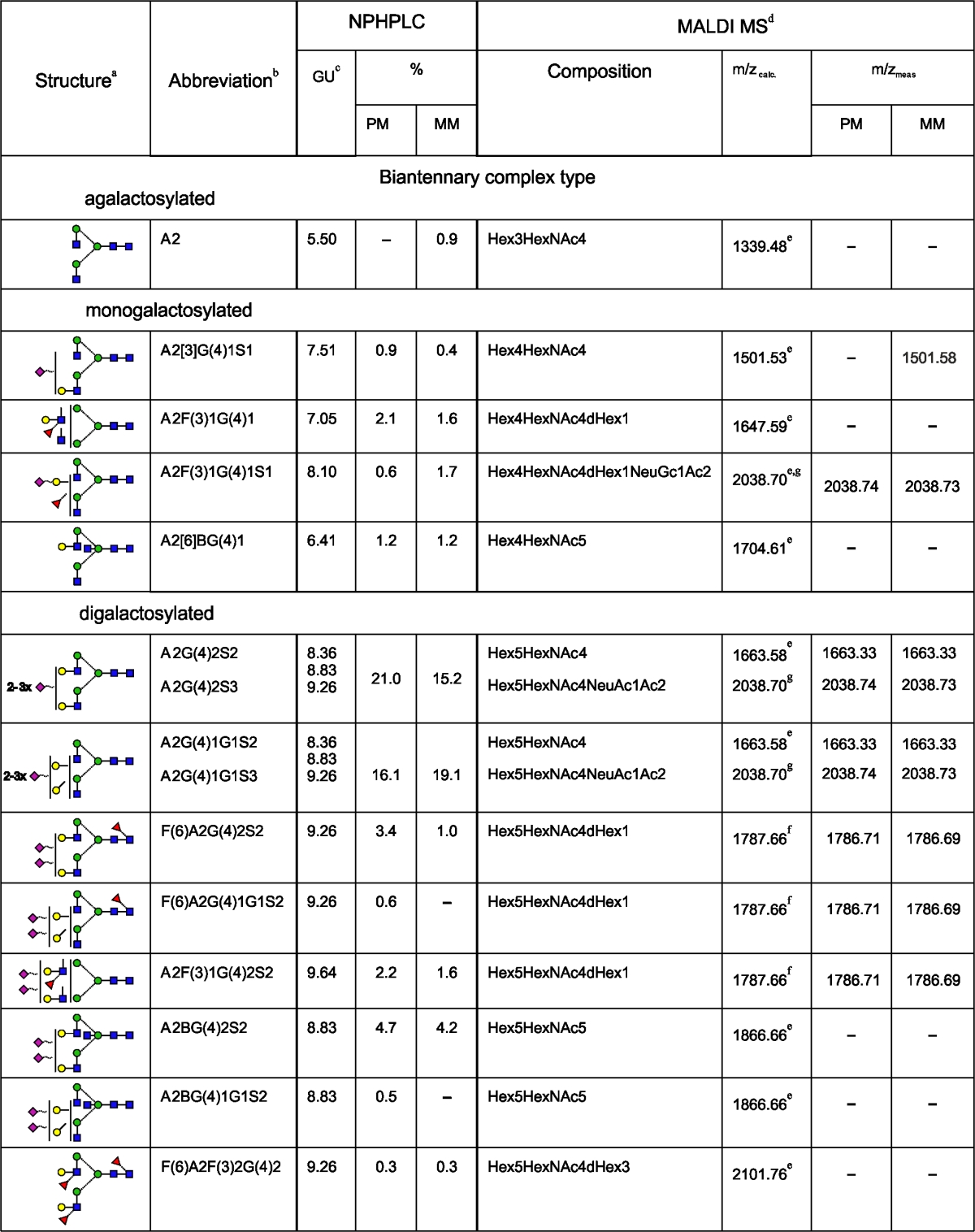

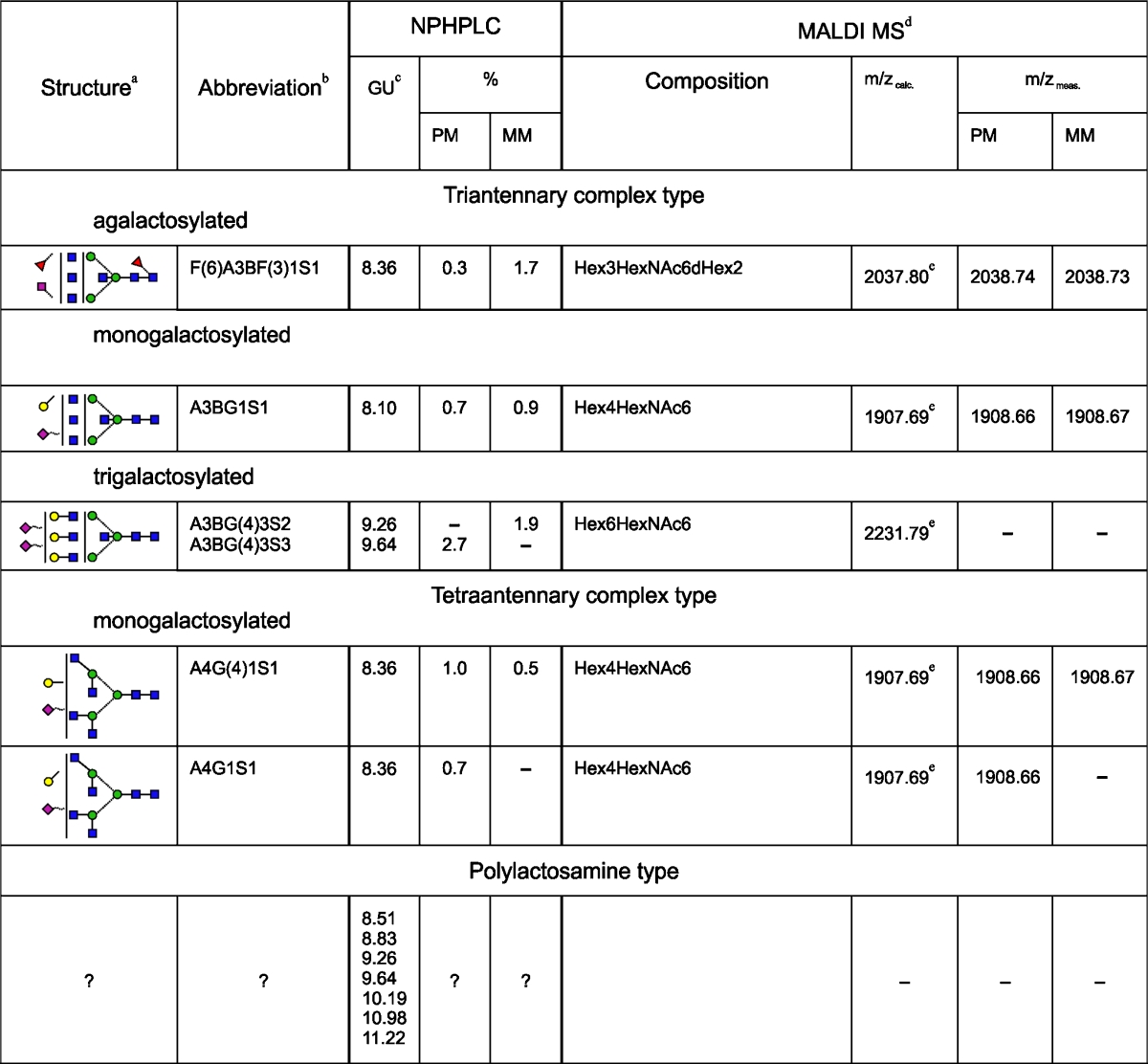
N-linked oligosaccharides in primary and metastatic melanoma L1CAM

PM, data for oligosaccharides of L1CAM from primary melanoma cells; MM, data for oligosaccharides of L1CAM from metastatic melanoma cells; +, the presence of corresponding structure in L1CAM; −, the absence of corresponding structure in L1CAM

^a^Symbol representation of glycans as follows:, GlcNAc;, mannose;, galactose;, fucose;, sialic acid; beta linkage, solid line; alpha linkage, dotted line; 1-4 linkage, horizontal line; 1-3 linkage, (/); 1-2 linkage, vertical line; and 1-6 linkage, (\)

^b^Structure abbreviations were used as in Fig. 2

^c^The corresponding structure is coeluted with a structure of similar GU except structures: M3, A1[6], F(6)A1 and M3, A1[6] in case of L1CAM from primary and metastatic melanoma cells, respectively

^d^Data for desialylated oligosaccharides

^e^[M+Na]+

^f^[M+H]+

^g^Structure resulted from partial digestion

No determination of the sialic acid linkage can be drawn from NPHPLC and MALDI MS data

## Lectin probes

**Fig. 3:**
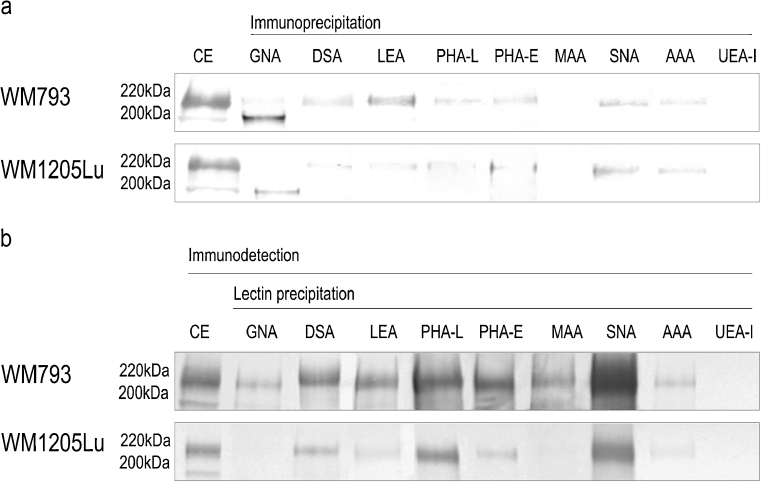
Lectin- and Western-blot analysis of L1CAM glycosylation. Immunoprecipitated L1CAM from primary WM793 and metastatic WM1205Lu cell lines was probed with a set of lectins **a**. Immunodetection of L1CAM from primary WM793 and metastatic WM1205Lu cell lines in lectin precipitates, using anti-L1CAM mAb (UJ127.11; 1: 12500) **b**. Lectin abbreviations and binding specificities are as follows: GNA, *Galanthus nivalis* agglutinin (recognises high mannose and hybrid-type oligosaccharides); DSA, *Datura stramonium* agglutinin (recognises two or three *N-*acetyllactosaminyl repeats); LEA, *Lycopersicon esculentum* agglutinin (recognises three or more *N-*acetyllactosaminyl repeats); PHA-L, *Phaseolus vulgaris* leucoagglutinin (recognises GlcNAcβ1-6-branched triantennary and tetraantennary complex-type glycans); PHA-E, *Phaseolus vulgaris* erythroagglutinin (recognises bisecting GlcNAc residue); MAA, *Maakia amurensis* agglutinin (recognises sialic acid α2-3-bound to terminal galactose residue); SNA, *Sambucus nigra* agglutinin (recognises sialic acid α2-6-bound to terminal galactose); AAA, *Aleuria aurantia* agglutinin (recognises fucose residues); UEA-I, *Ulex europaeus* I agglutinin (recognises fucose α1-2-bound to terminal galactose residue). CE, immunodetection of L1CAM in whole cell extract

**Fig. 4:**
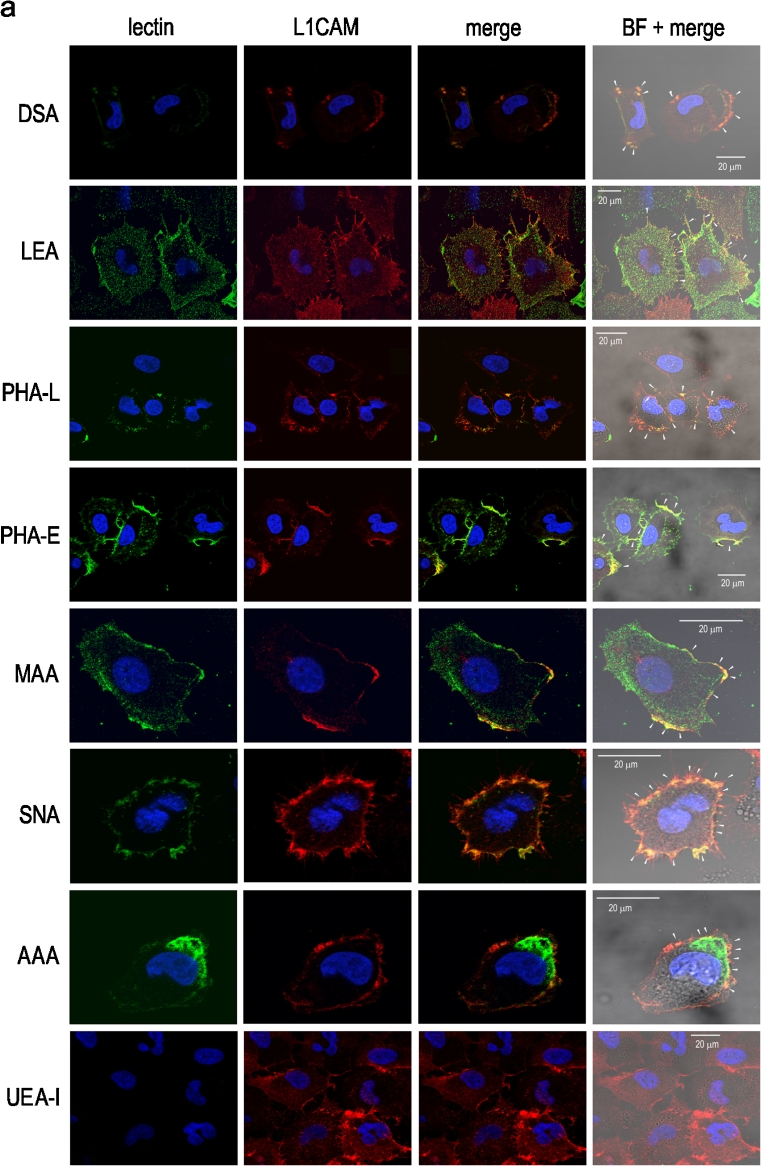

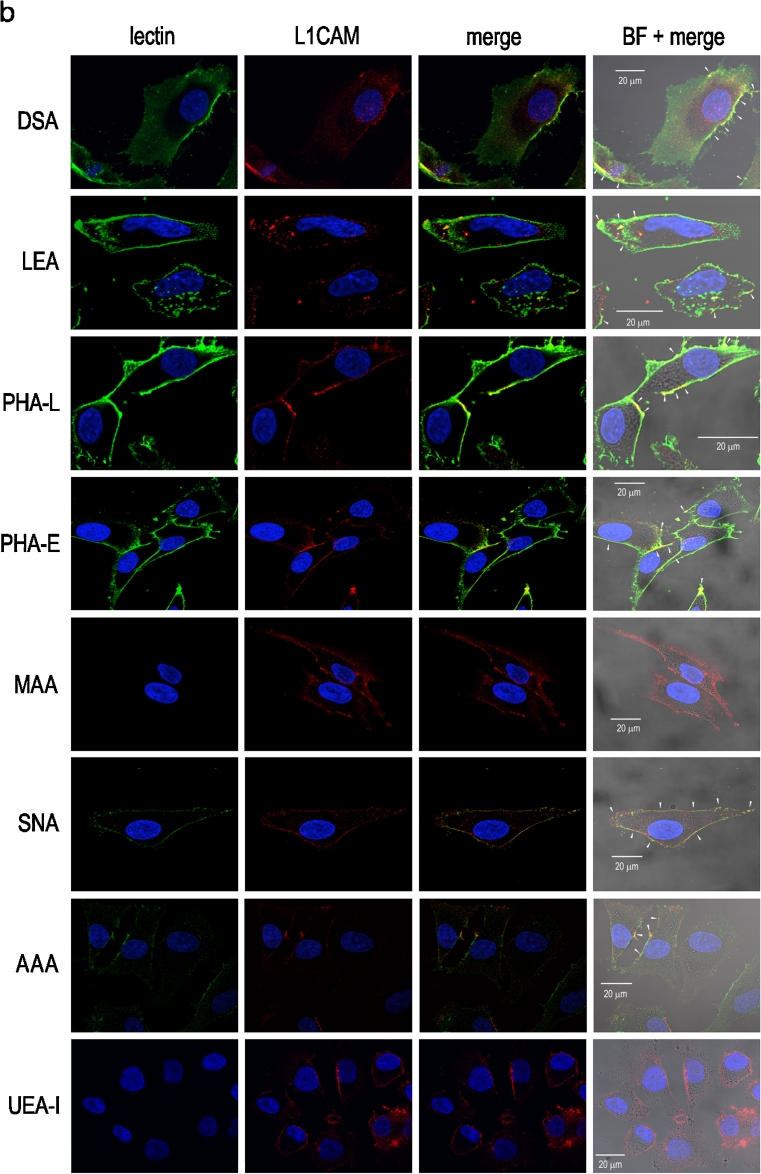
Confocal microscopy images of doubly labelled primary WM793 and metastatic WM1205Lu cells. Primary **a** and metastatic **b** melanoma cells were stained with a given lectin (*green*) and with anti-L1CAM antibody (*red*). Overlapping of the two stains (third and fourth panels), observed as *yellow* regions (white arrows), confirmed the presence of specific glycan residues in L1CAM molecules. Nuclei were counterstained with DAPI (*blue*). Lectin abbreviations and binding specificities as in Fig. 4. BF, bright field

To confirm the HPLC-identified structures we used a set of lectins to examine the L1CAM oligosaccharides. Immunoprecipitation followed by *on blot* lectin probing (Fig. 3a) and lectin precipitation followed by *on blot* immunodetection of primary and metastatic L1CAM (Fig. 3b) showed bands of two different weights, 200 and 220 kDa depending on the type of *N-*glycosylation. The bands of lower molecular weight showed a positive reaction with GNA (Fig. 3a). These bands corresponded to premature forms of L1CAM molecules, as they were exclusively GNA-positive, meaning that the premature form of L1CAM possessed only high mannose or high mannose and hybrid-type oligosaccharides. When lectin precipitation was used, both lower-molecular bands showed no GNA-positive reaction (Fig. 3b). It is likely that the high-mannose chains of the premature forms were buried within the protein folds and made inaccessible to GNA lectin capture. In the case of primary L1CAM, the band of higher molecular weight was GNA-positive. The bands of higher molecular weight also gave positive reactions with other lectins. The DSA- and LEA-positive bands confirmed the presence of poly-*N-*acetyllactosamine species in L1CAM. LEA binds oligosaccharides containing at least one side chain, preferentially the C-6 side, with three or more *N-*acetyllactosaminyl repeats. On the other hand, DSA binds oligosaccharides with shorter poly-*N-*acetyllactosaminyl units (two or three *N-*acetyllactosaminyl repeats) attached to C-2 and 6 of α-mannose on the C-6 side. However, LEA and DSA do not bind triantennary oligosaccharides with 2,4-substituted α-mannose and 2-substituted α-mannose by poly-*N-*acetyllactosaminyl units [27]. PHA-L- and PHA-E-positive staining confirmed the presence of GlcNAcβ1-6-branched triantennary and/or tetraantennary complex-type glycans and the presence of bisecting GlcNAc bound to the core mannose of complex *N-*glycans, respectively. A positive reaction with SNA showed the presence of α2-6-linked sialic acid. The altered reactivity of primary and metastatic L1CAM with MAA showed the presence of α2-3-linked sialic acid only in L1CAM from primary melanoma (Fig. 3b). For primary L1CAM we did not observe an *on blot* MAA-positive reaction after immunoprecipitation (Fig. 3a). This might be explained by low abundance of α2-3-linked sialic acid and its relative preferential loss due to sample boiling before electrophoresis. The presence of fucose and monosaccharide residue to which fucose had been bound was revealed in reactions with AAA and UEA-I lectins. AAA binds Fucα1-6 linked to the proximal GlcNAc residue as well as Fucα1-2Galβ1-4GlcNAc sequence (blood group H(0) determinant) and Galβ1-4(Fucα1-3)GlcNAc sequence (Le^x^ determinant). On the other hand, UEA-I recognises only Fucα1-2 linked to Gal. Both bands of higher molecular weight showed negative reactions with UEA-I and a positive reaction with AAA lectin (Fig. 3). These results supported the presence of core fucose and Le^x^ structure in primary and metastatic L1CAM. Confocal microscopy yielded the same results (Fig. 4). The confocal microscope images of cells doubly labelled, first with a given lectin and then with anti-L1CAM antibody, showed overlapping of the two stains, observed as yellow regions, and confirmed the presence of specific glycan residues in L1CAM molecules. Overlapping was not observed with UEA-I lectin staining, suggesting the absence of Fuc α1-2-linked to Gal residue. The presence of sialic acid on the cell surface was revealed using MAA and SNA lectins. In primary cells, MAA and SNA lectins showed moderate and strong staining, confirming the presence of α2-3- and α2-6-bound sialic acid, respectively. Unlike L1CAM from primary cells, L1CAM from metastatic cells was MAA-negative. Staining with GNA is not shown because there was no positive reaction although the staining was repeated three times. As mentioned before, this discrepancy might be explained by the inaccessibility of high-mannose chains to GNA lectin staining. The only observed difference in glycosylation patterns between metastatic L1CAM and L1CAM from primary cells was the absence of α2-3-linked sialic acid in the former. We wanted to find out whether that is a more general hallmark of L1CAM glycosylation during the transition of melanoma cells from the vertical growth phase to a metastatic stage. To answer this we precipitated glycoproteins from four primary and three metastatic cell lines using MAA and SNA lectins, and determined that the loss of α2-3-linked sialic acid in L1CAM is a phenomenon observed during the transition of melanoma cells from VGP to a metastatic stage (Supplementary Fig. S2).

## Mass spectrometry

We used mass spectrometry to complement the HPLC analysis of glycans. Since the amount of material available from each chromatographic peak was not sufficient for mass spectrometric analysis, we analysed total glycan pools using MALDI-TOF MS. Beforehand we had enzymatically desialylated both samples to simplify the spectra and thereby achieve a satisfactory signal-to-noise ratio. Interestingly, even after prolonged (>24 h) digestion with Sialidase A, which is known to cleave terminal and branched sialic acid, Neu5Ac/Neu5Gc desialylation appeared to be more complete than removal of acetylated Neu5Ac/Neu5Gc. These data are in agreement with earlier studies on the specificity of neuraminidases against *O*-acetylated sialoglycoconjugates [28]. It has been shown that human cancers, including melanoma, can take up and metabolically incorporate the non-human sialic acid Neu5Gc from dietary sources (or from fetal bovine serum in the case of cultured human cancer cells). Moreover, lysates of human melanoma cells had been found to exhibit *O*-acetyltransferase activity capable of generating the *O*-acetylated sialoglycoconjugates [29]. The MALDI-MS spectra showed four and six different pseudomolecular ions for primary and metastatic glycan pools, respectively. Among them were two abundant peaks: at m/z 1663.33 [M+Na]^+^ corresponding to Hex5HexNAc4 composition, and at m/z 1786.68 [M+H]^+^ corresponding to Hex5HexNAc3NeuGc1Ac1 and Hex5HexNAc4dHex1 compositions. The ion at m/z 1908.67 [M+Na]^+^ corresponded to Hex6HexNAc3NeuGc1 and Hex4HexNAc6 compositions. For the ion at m/z 2038.73 [M+Na/H]^+^ the following predicted compositions are calculated: Hex4HexNAc5NeuAc1Ac1, Hex6HexNAc3dHex1NeuAc1, Hex3HexNAc6dHex2, Hex5HexNAc4NeuAc1Ac2 and Hex4HexNAc4dHex1NeuGc1Ac2. Two additional ions at m/z 1296.51 [M+Na]^+^ corresponding to Hex4HexNAc3 composition and at m/z 1501.53 [M+Na]^+^ corresponding to Hex4HexNAc4 composition were observed in the sample from metastatic cells (Supplementary Fig. S3). The corresponding candidate structures were juxtaposed with the structures resulting from HPLC analysis (Table 1). Some oligosaccharides found by HPLC were not confirmed by MALDI MS. Using ESI-FT-ICR we confirmed the presence of HexNAc1NeuGc1 and/or Hex1NeuGc1 (^3,5^A_HexNAc_ and/or ^3,5^A_Hex_ ions at m/z 382.135) and the presence of Hex2NeuAc1 and/or Hex2NeuGc1 (^1,5^XC_NeuAc_ and/or ^1,5^XC_NeuGc_ ions at m/z 415.107; fragment ions confirming the presence of the novel structure) with mass accuracy below 2.4 ppm (data not shown).

## Functional studies

**Fig. 5:**
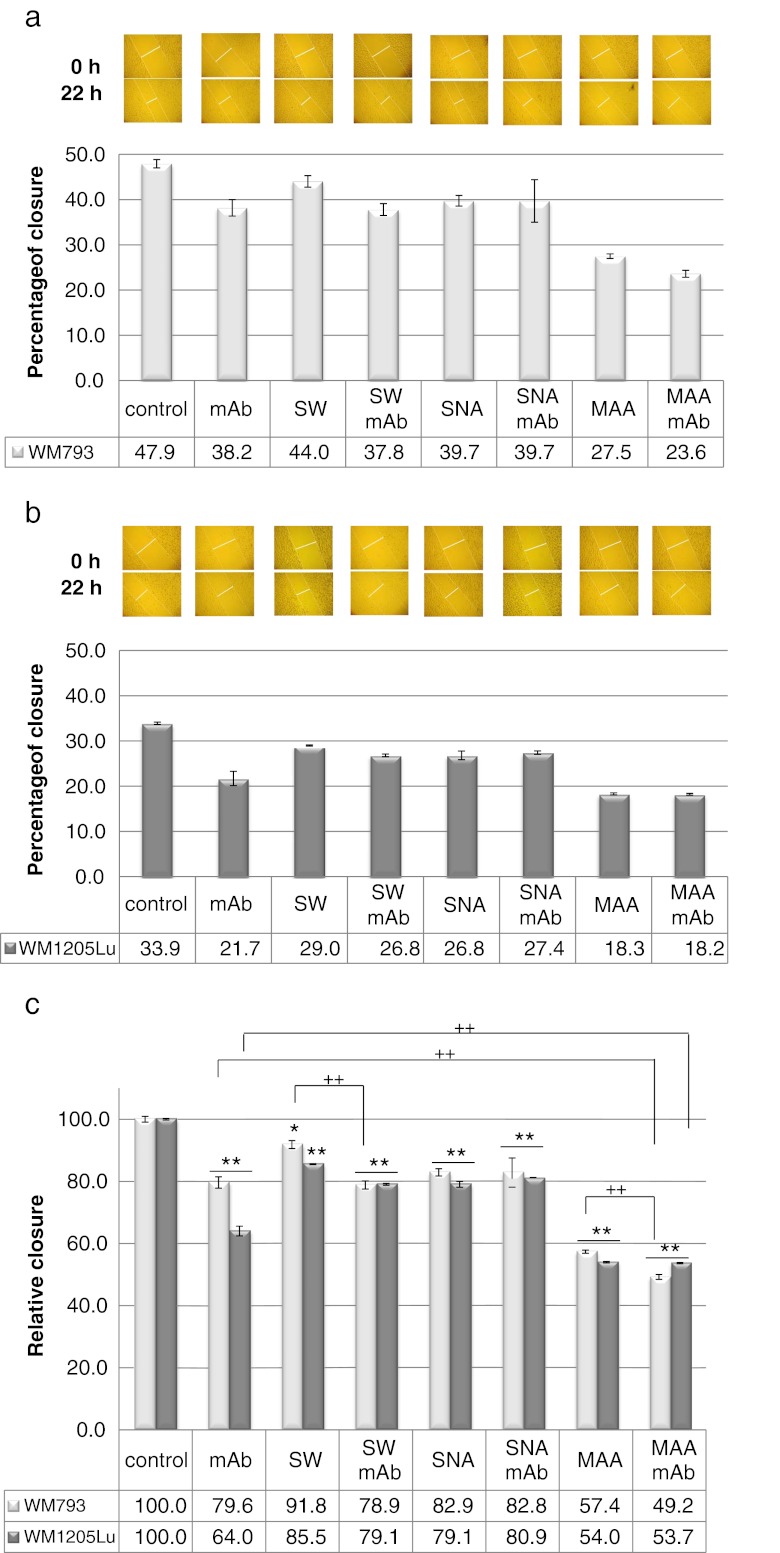
Effect of cell surface oligosaccharides and L1CAM oligosaccharides on repair of wounds in monolayers of primary WM793 and metastatic WM1205Lu melanoma cells. The cell-coated surface was scraped with a plastic tip in a single stripe and covered with medium supplemented with 10 % FCS. The wounds were allowed to heal without (control) or in the presence of anti-L1CAM antibody (mAb; 10 μg/ml), swainsonine (SW; 10 μl/ml), MAA (50 μg/ml), SNA (50 μg/ml), both anti-L1CAM mAb and SW, or both anti-L1CAM mAb and lectin. The wounded areas were photographed immediately after the cell monolayers scrape-wounded (0 h) and after 22 h. The average extent of wound closure for primary **a** and metastatic **b** cells was quantified from twenty measurements of the wound space width in two separate trials for each treatment using AxioVision software (Carl Zeiss). Values are means ± standard deviation of three separate experiments (*bars*). **c** Changes in cell migration rate after antibody, lectin and SW treatment were calculated by comparing the migration of untreated (control; taken as 100 % migration) and treated cells. Lectin abbreviations and binding specificities as in Fig. 4. *P < 0.05 and **P < 0.01 *vs.* suitable control; ++*P* < 0.01 (Duncan’s *t* test)

The wound healing studies were intended to evaluate the role of cell surface oligosaccharides and L1CAM oligosaccharides in the migratory behaviour of melanoma cells. The wounds were allowed to heal in the presence of L1CAM mAb, SW (a potent inhibitor of complex *N-*type glycan processing), MAA, SNA, both L1CAM mAb and SW, or both L1CAM mAb and lectin. Apparently, primary melanoma cells closed the wound faster than metastatic cells did. The primary cells were much flatter than the metastatic cells. It was very difficult to see the cell edge with an inverted microscope (Fig. 5a and b). Basal migration was normalised to 100 % and the relative closure of wounds was calculated (Fig. 5c). In primary cells, mAb alone inhibited migration more than was seen under treatment with SW alone, and combined mAb and SW treatment did not result in further inhibition of primary cell motility. The effects on primary cell motility were similar for the set of treatments applying mAb alone, SNA alone and combined SNA and mAb. It seems that the antibody treatment rather than SW or SNA treatment influenced the motility of primary cells. Interestingly, MAA treatment strongly inhibited primary cell motility, and combined MAA and mAb treatment resulted in less than additive inhibition, suggesting a significant role for α2-3-sialylated L1CAM in this process. Immunoinhibition by L1CAM or SW treatment resulted in stronger inhibition of cell motility in metastatic cells than in primary cells. These findings suggest a more important role for L1CAM and the more obvious role of complex-type *N-*glycans of metastatic cells in wound healing. Combined SW and mAb treatment had no additive effect. SNA alone or in concert with mAb had the same effect as observed in primary cells. Surprisingly, MAA treatment inhibited metastatic cell motility by 46 %. Although the surfaces of both types of cells are α2-3-sialylated (Supplementary Fig. S4), the combined MAA and mAb treatment did not change the level of inhibition in the case of metastatic melanoma cells. As we showed above, metastatic L1CAM did not possess α2-3-linked sialic acid.

**Fig. 6:**
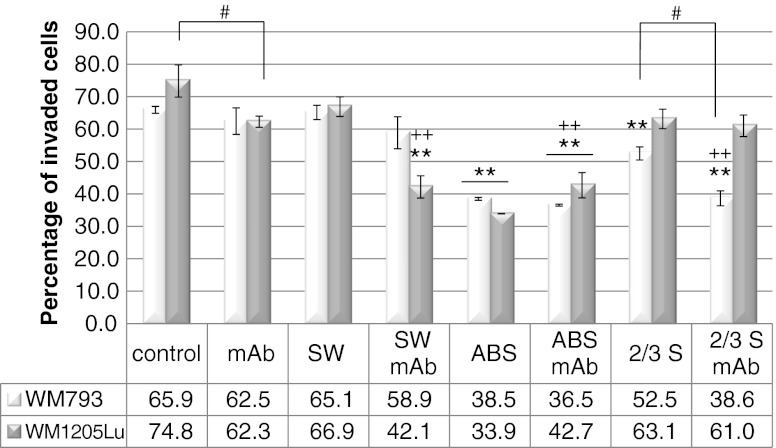
Effects of L1CAM, complex *N-*glycans and sialic acid on the invasiveness of primary WM793 and metastatic WM1205Lu melanoma cell lines in a matrigel invasion assay. Fluorescence-labelled cells were seeded on top of either uncoated or matrigel-coated filters. To promote invasion the lower part of the chamber was filled with 5 % FBS. For some experiments, before invasion assays the cells were grown in the presence of swainsonine (SW; 10 μg/ml) and then the tumour cells were seeded into the wells either in the presence of SW (10 μg/ml) or in the presence of SW and anti-L1CAM antibody (mAb; 40 μg/ml). For some experiments, before the invasion assays the cells were desialylated with neuraminidases from *Arthrobacter ureafaciens* (broad specificity) or *Streptococcus pneumoniae* (removes α2,3 linked sialic acid) and then tumour cells were incubated in the presence or absence of anti-L1CAM antibody (40 μg/ml) for another 1 h. Finally, the tumour cells were seeded into the wells. After 17 h incubation the fluorescence of cells in lower chambers was measured with a BioTek Synergy™ instrument. The percentage of cell invasion was calculated as follows: *Formula*: % invasion = (Mean RFU of cells that invaded through matrigel-coated membrane/Mean RFU of cells that migrated through uncoated membrane) * 100. Control was untreated cells. Each experiment was done twice or three times, with four repetitions for each sample. Values are means ± standard deviation (*bars*). **P < 0.01 *vs.* suitable control; ++*P* < 0.01 *vs.* suitable mAb-treated cells; # *P* < 0.05 (Duncan’s *t* test)

Next we examined whether cell surface oligosaccharides and L1CAM oligosaccharides could influence the invasiveness of melanoma cell lines in a matrigel invasion assay. We looked at the effects of L1CAM mAb, SW, treatment with *A. ureafaciens* sialidase or *S. pneumoniae* sialidase (the latter hydrolyses non-reducing terminal α2-3-linked sialic acid from complex glycans and glycoproteins), as well as treatment with both L1CAM mAb and SW or both L1CAM mAb and sialidase. It should be noted that both cell lines showed comparable invasiveness (Fig. 6). Antibody treatment reduced the invasive potential of primary and metastatic cells by 6 % and 18 % respectively. SW treatment had no effect on the invasive potential of the primary cell line and a very low or no effect on the invasiveness of the metastatic cell line. Treatment with both L1CAM mAb and SW caused a slight additional reduction of the invasive potential of the primary cell line and exerted a synergistic effect in the metastatic cell line (40 % reduction). This synergistic effect suggests an important role for L1CAM complex-type glycans in the invasive behaviour of metastatic cells. ABS treatment strongly inhibited the invasiveness of both cell lines (more in metastatic than in primary cells). In the combined treatment with ABS and L1CAM mAb, additional inhibition of invasiveness was observed only in primary cells. The removal of α2-3-linked sialic acid from the cell surface of primary cells caused 20 % inhibition of the invasiveness of these cells, and negligible inhibition in metastatic cells. Combined treatment with *S. pneumoniae* sialidase and mAb showed an additional effect, suggesting the important role of α2-3-linked sialic acid in the L1CAM molecule in the invasive behaviour of primary melanoma cells. As demonstrated above, L1CAM of primary melanoma but not L1CAM of metastatic melanoma possesses α2-3-linked sialic acid.

Untreated, SW-treated, ABS-treated and *S. pneumoniae* sialidase-treated cells were collected, protein cell extracts were prepared, and the effects of treatment were examined by western blotting (Supplementary Fig. S5). SW and ABS treatments shifted the molecular weight of L1CAM to a lower value in primary and metastatic cells. *S. pneumonia* sialidase treatment caused a negligible or no shift of its molecular weight to a lower value in primary cells. This can be explained by the low level of α2-3-linked sialic acid in the primary L1CAM molecule.

Our findings confirm the previous observation that L1CAM affects migratory behaviour and invasiveness more strongly in metastatic than in primary melanoma cells [24]. L1CAM from VGP melanoma cells and metastatic cells showed quite similar glycosylation patterns. This suggests that oncogenic changes in glycan biosynthesis are established in the early stages of melanoma transformation. The loss of α2-3-linked sialic acid in L1CAM is a phenomenon observed during the transition of melanoma cells from VGP to a metastatic stage. The absence of α2-3-sialylated structures might affect the synthesis of sialyl Le^x^ in L1CAM of metastatic cells. Possibly the cells from metastatic side no longer needed the sialylated form of Le^x^. The presence of cell surface α2-3-linked sialic acid facilitates the migratory behaviour and increases the invasiveness of primary melanoma cells, and it enhances the motility of metastatic cells. The presence of cell surface α2-6-linked sialic acid enhances the invasive potential of both primary (VGP) and metastatic melanoma cells. Complex-type oligosaccharides in L1CAM enhance the invasiveness of metastatic melanoma cells.

## Discussion

Our results demonstrate that human melanoma L1CAM accumulates cancer-associated glycan structures, among which are β1-6-branched complex-type glycans with or without *N*-acetyllactosamine repeats, fucose α1-3-linked to GlcNAc in the antenna (Le^x^), low-mannose structures, as well as GlcNAc-terminated complex-type glycans. Previously, it was shown that β1-6-branched oligosaccharides are not produced by melanocytes or by cells of early melanoma *in situ* but are characteristic structures of florid melanoma *in situ*, as well as invasive and metastatic tumours [30]. A marked and progressive increase of these oligosaccharides during the transformation process has been demonstrated [31]. Melanoma cells with a higher level of β1-6-branched oligosaccharides displayed a greater ability to metastasize to lungs and produced larger colonies [5]. The β1-6-branched *N-*glycans extended with poly-*N-*acetyllactosamine chains facilitate lung-specific metastasis of melanoma cells *via* galectin-3 [7]. Normal human melanocytes did not express sLe^x^ and poorly expressed sLe^a^, but melanoma cells from tumour biopsies and culture overexpressed both isomers; sLe^x^ and sLe^a^ were associated with glycoprotein and glycolipid, respectively [32]. These findings suggested that sLe^x^ and sLe^a^ are neoplastic differentiation antigens of melanoma, which are potentially recognised by selectins and thus may facilitate tumour progression and metastasis. The metastatic capability of human melanoma MeWo cells and mouse melanoma B16 cells dramatically increased after acquiring sLe^x^ through transfection of α1,3-fucosyltransferase III (FucT-III) [33, 34]. Recently, the expression of two other cancer-associated *N-*glycans was detected in several malignant tumours, including melanoma and its liver metastases: core fucosylated (Man_1-5_GlcNAc2Fuc1) and non-fucosylated (Man_1-4_GlcNAc2) low-mannose-type and terminal GlcNAc-structures [35, 36]. Man3GlcNAc2 (Table 1) is expressed on many glycoproteins of invertebrate organisms, and to a limited extent on mammalian glycoproteins [35]. Similarly, terminal GlcNAc antigens corresponding to Hex3HexNAc3dHex_0-1_ and Hex3HexNAc4 compositions (Table 1) are very rare on the surface of normal human cells [36]. On the other hand, malignant human tumours express considerable amounts of abnormal terminal GlcNAc in protein-linked glycans. These glycans are specifically accumulated in lung tumour [36]. Curiously, our data unambiguously defined a novel *N-*glycan structure containing di- and trisialylated Galβ1-4Galβ1-4GlcNAc sequence on the 3-arm antenna of monoantennary complex-type glycan. This result was surprising, because nothing is presently known about the possible expression of Galβ1-4Galβ1-4GlcNAc sequence on either glycoproteins or glycolipids in man or other mammals. The Galβ1-4Galβ1-4GlcNAc motif monosialylated with either Neu5Gc or Neu5Ac and/or fucosylated has been identified in eggs of several fish species [37–39]. A slightly different version with additional α4-Gal capping instead of sialylation or fucosylation has also been identified on pigeon serum immunoglobulin G and in O-linked glycans of salivary gland mucin glycoproteins of the Chinese swiftlet [40, 41]. The uncovered Galβ1–4Galβ1- motif was found on a glycolipid isolated from ostrich liver [42]. No clear function has yet been assigned to Galβ1–4Galβ1- in animal glycoproteins, but it was demonstrated that Galβ1–4Galβ1- at non-reducing termini was strongly antigenic, causing “pigeon fancier’s lung”, a form of extrinsic allergic alveolitis in man [43]. Malignancy and metastasis are associated with an overall increase in cell-surface sialic acid content, which in turn reduces the attachment of metastatic tumour cells to matrix components and thereby promotes and facilitates the migration of benign and malignant melanocytes. Sialic acids facilitate the association of malignant cells with selectins, causing interactions of circulating tumour cells with platelets, leukocytes and the endothelium, facilitating metastasis [44]. It is possible that altered sialylation of tumour cells affects interactions with some Siglecs found on innate immune cells [44]. An increase in sialylation is often manifested as a specific increase in α2-6-linked sialic acid attached to outer *N-*acetyllactosamine. Apart from changes in the amount and linkage of sialic acid, there can also be significant modifications. It has been documented that sialic acid 9-*O*-acetylation of gangliosides increased in melanoma cells. 9-*O*-acetylneuraminic acid has been recognised as a specific marker for human melanoma cells [29, 45]. Normal human adult melanocytes express a high amount of Neu5Ac sialic acid, but they do not express acetylated forms of sialic acids. Since this modification by *O*-acetylation could make cells much less susceptible to degradation and extend their lifetime *in vivo*, it could be related to the high malignancy and rapid spread of human melanoma [46]. Another interesting phenomenon is the aberrant expression of Neu5Gc (HD antigen) in human cancer cells [47, 48]. More conspicuous enrichment of Neu5Gc in carcinomas could be caused by higher uptake by these rapidly growing tissues [49]. Here we have shown that L1CAM protein can also carry an acetylated form of sialic acid in human melanoma. To our knowledge, A1[3]G(4)2 structure [Galβ1-4Galβ1-4GlcNAcβ1-2Manα1-3(Manα1-6)Manβ1-4GlcNAcβ1GlcNAc] of L1CAM acetylated on sialic acid is the first cancer biomarker related to metabolic replacement of Neu5Ac with the immunogenic dietary NeuGc molecule in protein-linked *N-*glycans. The coexistence of acetylated *N-*glycolylneuraminic acid with Galβ1-4Galβ1-4GlcNAc sequence in A1[3]G(4)2 structure generated a novel, unique neo-tumour-associated xeno-autoantigen in human melanoma. The appearance (or elevated amounts as compared with healthy cells) of any one of the above mentioned cancer-related oligosaccharide sequences on the cell surface indicates the cancerous nature of the cells or tissues. This report is the first to describe simultaneous expression of all the above-mentioned cancer-related antigens in L1CAM from human melanoma.

Tumour-specific expression and antibody recognition of the above-mentioned carbohydrate epitopes have been described for human IgM antibodies. IgM titers to sLe^x^ antigen are low in sera of normal individuals and high in sera of melanoma patients [32]. Normally, there are large amounts of antibodies recognizing terminal GlcNAc structures in human serum [50]. High titer of a natural antibody against terminal Galβ1–4Galβ1- was detected in human sera; the anti-Galβ1–4Galβ1- antibodies were mainly IgM and occurred in a large population [42]. Humans have varying and sometimes substantial levels of circulating antibodies directed against Neu5Gc antigen [51]. This antigen is known to trigger a potent immune reaction. In a human-like Neu5Gc-deficient mouse model, tumour-associated Neu5Gc interacted with low levels of circulating anti-Neu5Gc antibodies, thereby facilitating tumour progression *via* chronic inflammation and angiogenesis [52]. Thus, a weak immune response is considered to be favourable to tumour growth. Perhaps this is why the Galβ1–4Galβ1-4GlcNAc antigen presented in our study, an antigen potent enough to trigger a strong immune reaction, is masked by sialic acid. The acetylated form of sialic acid, more resistant to neuraminidase treatment, protects cells carrying these residues from apoptosis. Since cancer cells shed the L1CAM ectodomain into circulation, the NeuGcAcGalβ1-4Galβ1-4 xeno-autoantigen potentially gives us a novel serum biomarker for early detection of melanoma. This new structure presents an opportunity to identify other novel glycosyltransferase activities such as the galactosyltransferase activity involved in synthesis of the Galβ1-4Galβ1-4GlcNAc sequence. We are aware of the limitation of *in vitro* model, which bases on cells maintained in medium supplemented with foetal bovine serum– a source of NeuGc. Interpretation of cancer uptake of nonhuman NeuGc by melanoma cells must be integrated with clinical and histopathological studies to avoid misleading judgement.

## Conclusions

Here we showed for the first time that (i) the sialic acid linkage position in L1CAM could effectively discriminate between the VGP primary and the metastatic stage of human melanoma, and (ii) A1[3]G(4)2S_2-3_ oligosaccharide is a new melanoma-associated carbohydrate antigen, absent from the normal cell surface and resulting from alteration of *N-*glycan biosynthesis. Further work should more precisely determine the glycosylation pattern of L1CAM in different stages of melanoma progression, especially the diversity in sialic acid content. Also, the biological significance of Galβ1–4Galβ1- in human melanoma remains to be established. Whether Galβ1–4Galβ1- is located specifically on L1CAM glycoprotein or on glycoproteins of other tumours and cancer is another question to answer.

## Electronic supplementary material

Below is the link to the electronic supplementary material.

Fig. S1 Summary of the research design (PDF 56 kb)

Fig. S2 Western-blot analysis of L1CAM sialylation. Immunodetection of L1CAM in lectin precipitates from primary melanoma cell lines FM-55-P, IGR-39, WM1552C (RGP) and WM75 (VGP), and from metastatic melanoma cell lines M6/B7, UKRV-Mel-15a and Ma-Mel-27. The presence of sialic acid in L1CAM was revealed using MAA and SNA lectins. PHA-L and PHA-E lectins were used for comparison. Lectin abbreviations and binding specificities as in Fig. 4. CE, whole cell extract; PM, primary melanoma; RGP, radial growth phase melanoma; VGP, vertical growth phase melanoma; MM, metastatic melanoma; +, positive reaction with a given lectin; -, negative reaction with a given lectin. (JPEG 2.08 mb)

High resolution image file (TIFF 13.3 mb)

Fig. S2 Positive MALDI MS mass spectra of L1CAM oligosaccharides from primary WM793 and metastatic WM1205Lu cell lines. *N*-glycan pools were released from purified L1CAM by *in situ* digestion with PNGase F followed by desialylation by *A. ureafaciens* sialidase. Mass spectrum of L1CAM glycans from primary **a** and metastatic **b** melanoma cells. **c** The compositions deduced from mass values are given in the table. MM, metastatic melanoma; PM, primary melanoma; ^a^ [M+Na] + adduct ion; ^b^ [M+H] + adduct ion; ^c^ [M+K] + adduct ion; * Contaminating peak (JPEG 2.50 mb)

High resolution image file (TIFF 28.7 mb)

Fig. S4 Flow cytometric analyses of the expression of α2-3-linked sialic acid on the cell surface of primary WM793 **a** and metastatic WM1205Lu melanoma cells **b**. Briefly, cells (1 × 10^5^) were fixed with 4% PFA (10 min, 0 °C), incubated either with FITC-Avidin (1:100, 30 min, 0 °C) or with MAA-biotin (1.25:100, 30 min, 0 °C) followed by incubation with FITC-Avidin (1:100, 30 min, 0 °C). For some experiments, before cell fixation the cells were incubated with 50 mU/ml sialidase from *Arthrobacter ureafaciens* (broad specificity) (1 h, 37 °C). The cells were assessed for fluorescence in a FACSCalibur flow cytometer (BD Biosciences, San Diego, CA). A total of 10^4^ cells were analysed for each immunofluorescence profile. Shaded area, control cells treated with FITC-Avidin; solid line, FITC–MAA-stained cells; dotted line sialidase-treated cells followed by FITC–MAA-staining (PDF 29 kb)

Fig. S5 Assessment of effectiveness of swainsonine and sialidase treatment of WM793 and WM1205Lu melanoma cells. (UCE) Untreated, (SW) swainsonine-treated, (ABS) *A. ureafaciens* sialidase-treated and (2/3S) *S. pneumonia* sialidase-treated cells were collected, protein cell extracts were prepared, and the effectiveness of treatment was assessed by western blotting. (PDF 44 kb)

## Abbreviations

2AB: 2-Aminobenzamide
Ac: Acetyl group
dHex: Deoxyhexose
ESI-FT-ICR: Electrospray ionization Fourier transform ion cyclotron resonance
FBS: Fetal bovine serum
Hex: Hexose
HexNAc: *N*-Acetylhexosamine
LacNAc type 1: Galβ1-3GlcNAcβ-
LacNAc type 2: Galβ1-4GlcNAcβ-
Le^a^: Galβ1-3(Fucα1-4)GlcNAcβ-
Le^x^: Galβ1-4(Fucα1-3)GlcNAcβ-
MALDI-TOF: Matrix-assisted laser desorption/ionization
MS: Mass spectrometry
Neu5Ac: *N*-Acetyl-neuraminic acid
Neu5Gc: *N*-Glycolyl-neuraminic acid
NGS: Normal goat serum
NP HPLC: Normal phase high-performance liquid chromatography
RGP: Radial growth phase
TOF: Time-of-flight
VGP: Vertical growth phase
