# Supplementary figures and images for: L1CAM from human melanoma carries a novel type of N-glycan with Galβ1-4Galβ1- motif. Involvement of N-linked glycans in migratory and invasive behaviour of melanoma cells
Source: Glycoconj J. 2012 Apr 29;30(3):205–25. doi: 10.1007/s10719-012-9374-5 (PMC3606521; doi:10.1007/s10719-012-9374-5)

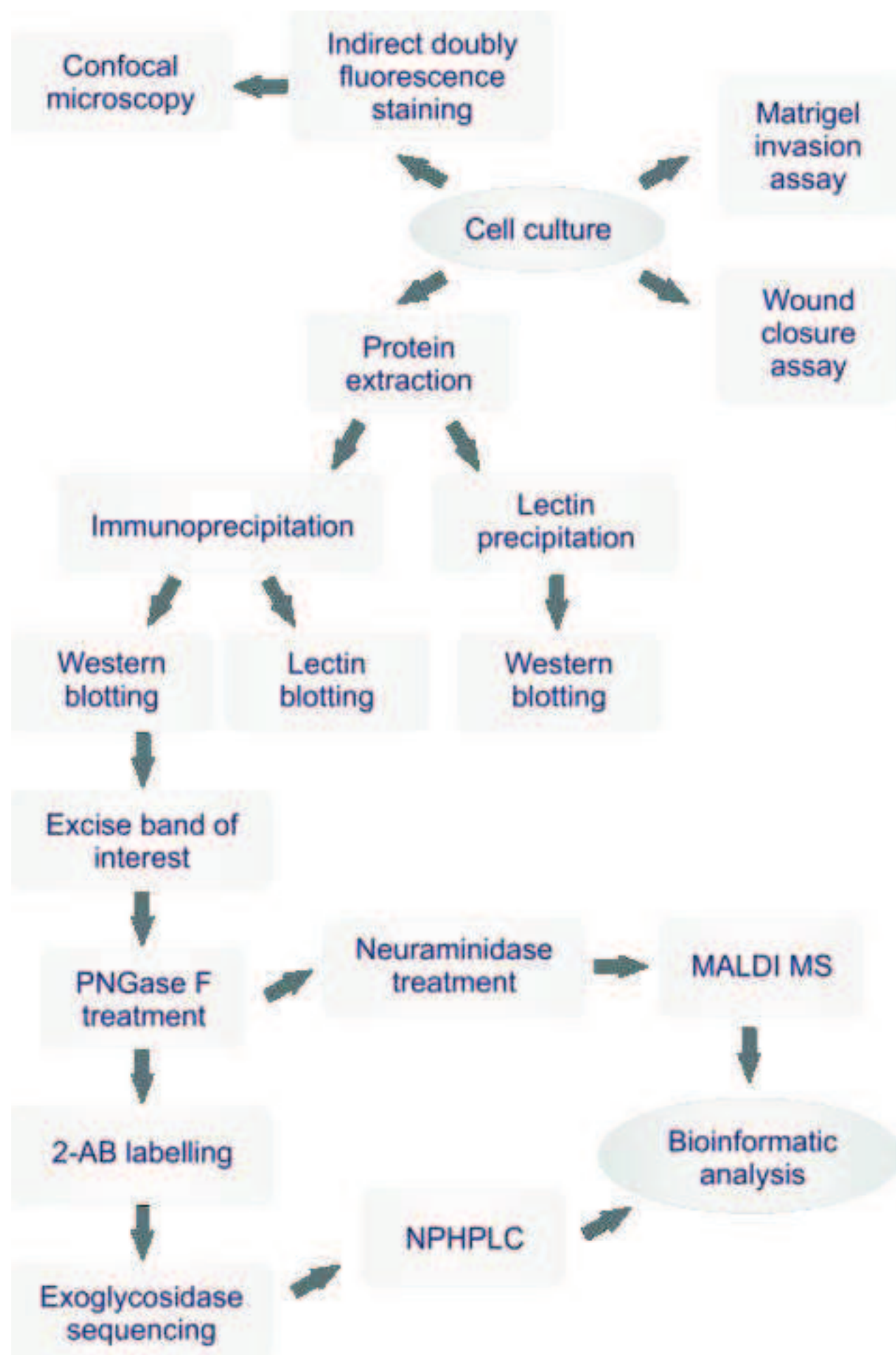

Supplement: Supplementary file 1 — Summary of the research design (PDF 56 kb) [file 10719_2012_9374_MOESM1_ESM.pdf]

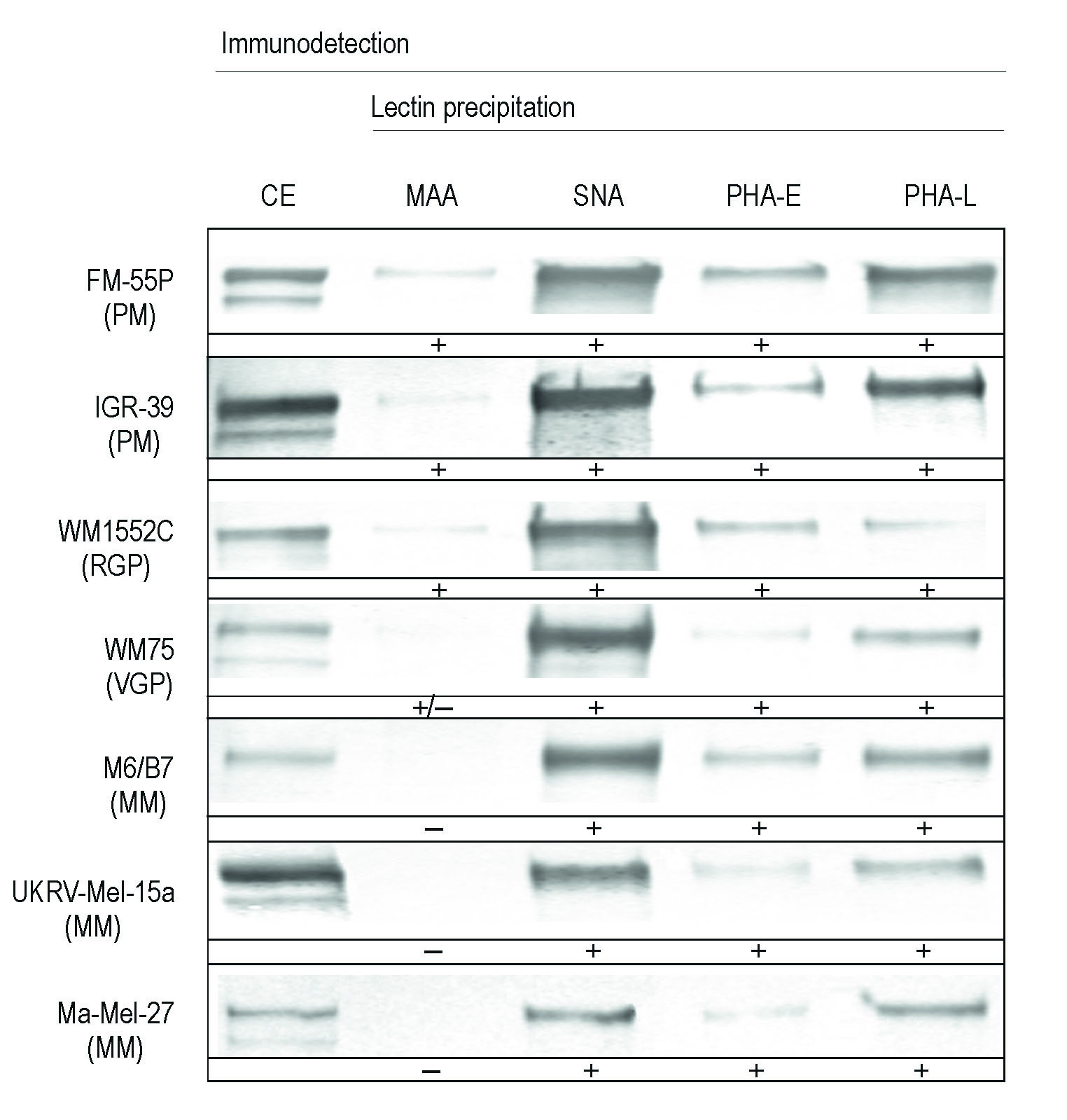

Supplement: Supplementary file 2 — Western-blot analysis of L1CAM sialylation. Immunodetection of L1CAM in lectin precipitates from primary melanoma cell lines FM-55-P, IGR-39, WM1552C (RGP) and WM75 (VGP), and from metastatic melanoma cell lines M6/B7, UKRV-Mel-15a and Ma-Mel-27. The presence of sialic acid in L1CAM was revealed using MAA and SNA lectins. PHA-L and PHA-E lectins were used for comparison. Lectin abbreviations and binding specificities as in Fig. 4. CE, whole cell extract; PM, primary melanoma; RGP, radial growth phase melanoma; VGP, vertical growth phase melanoma; MM, metastatic melanoma; +, positive reaction with a given lectin; -, negative reaction with a given lectin. (JPEG 2.08 mb) [file 10719_2012_9374_Fig7_ESM.jpg]

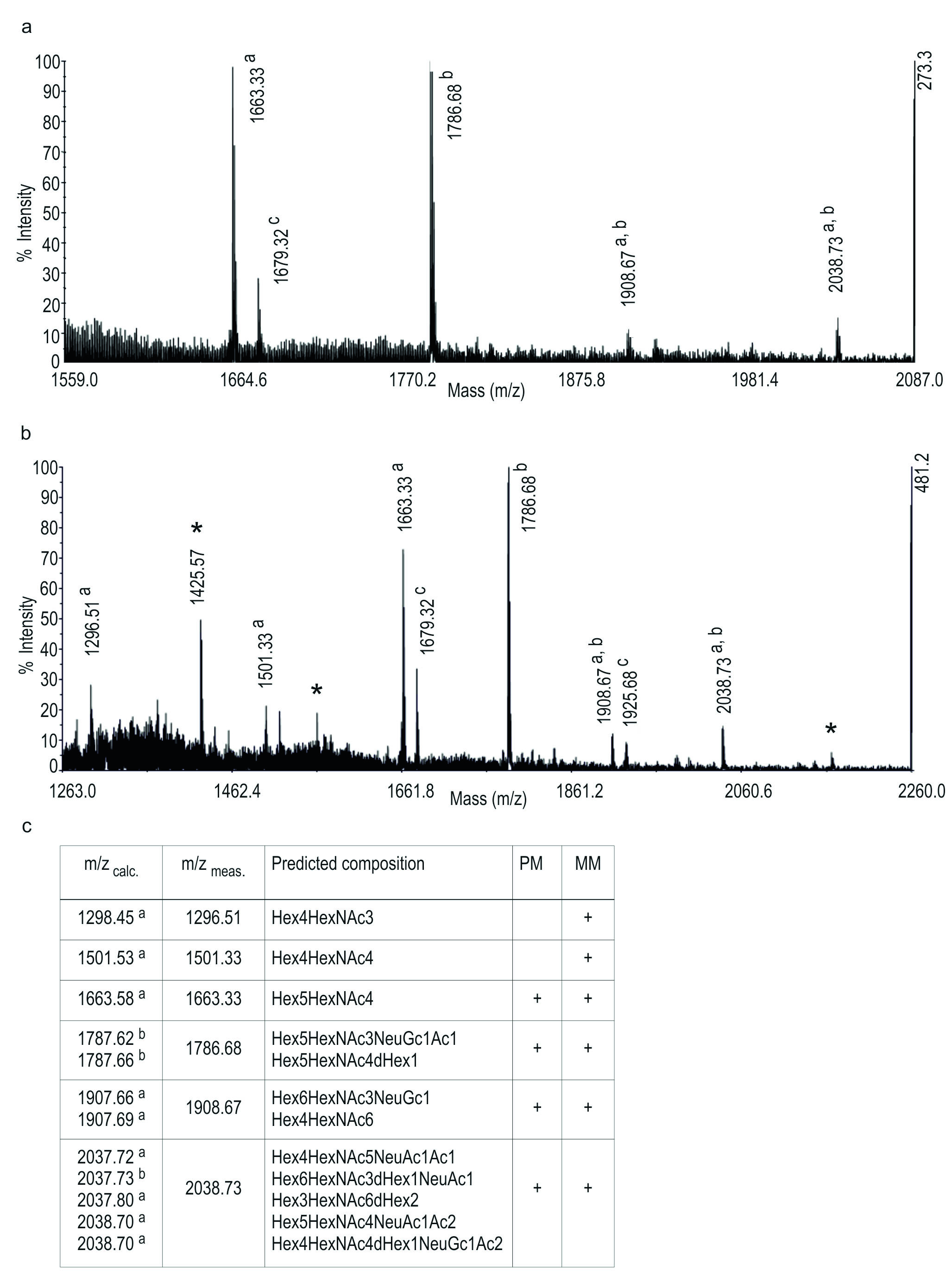

Supplement: Supplementary file 4 — Positive MALDI MS mass spectra of L1CAM oligosaccharides from primary WM793 and metastatic WM1205Lu cell lines. N-glycan pools were released from purified L1CAM by in situ digestion with PNGase F followed by desialylation by A. ureafaciens sialidase. Mass spectrum of L1CAM glycans from primary a and metastatic b melanoma cells. c The compositions deduced from mass values are given in the table. MM, metastatic melanoma; PM, primary melanoma; a [M+Na] + adduct ion; b [M+H] + adduct ion; c [M+K] + adduct ion; * Contaminating peak (JPEG 2.50 mb) [file 10719_2012_9374_Fig8_ESM.jpg]

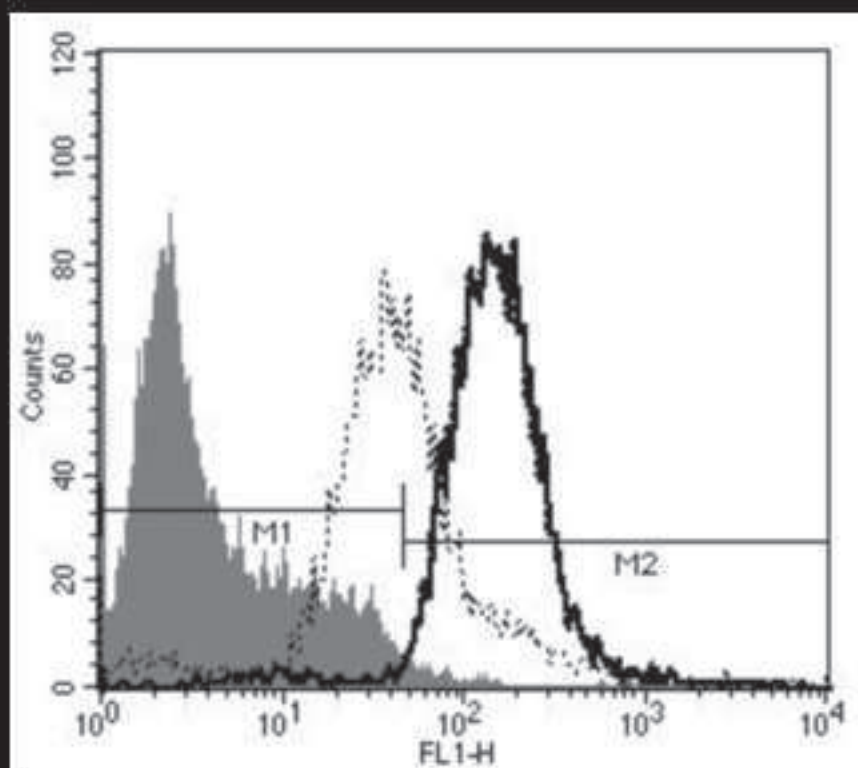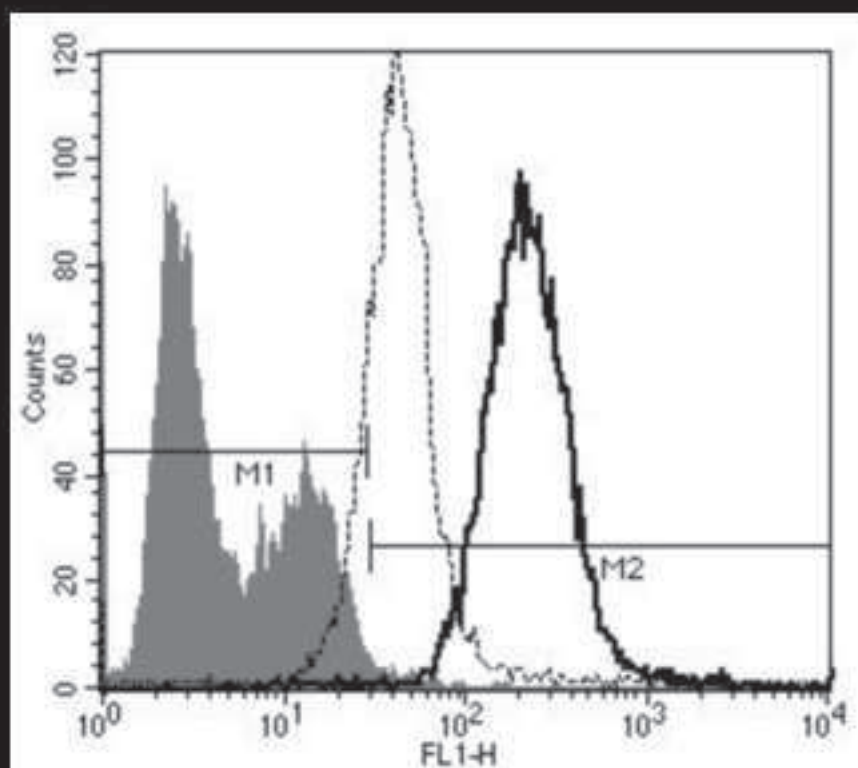

Supplement: Supplementary file 6 — Flow cytometric analyses of the expression of α2-3-linked sialic acid on the cell surface of primary WM793 a and metastatic WM1205Lu melanoma cells b. Briefly, cells (1 × 10^5^) were fixed with 4% PFA (10 min, 0 °C), incubated either with FITC-Avidin (1:100, 30 min, 0 °C) or with MAA-biotin (1.25:100, 30 min, 0 °C) followed by incubation with FITC-Avidin (1:100, 30 min, 0 °C). For some experiments, before cell fixation the cells were incubated with 50 mU/ml sialidase from Arthrobacter ureafaciens (broad specificity) (1 h, 37 °C). The cells were assessed for fluorescence in a FACSCalibur flow cytometer (BD Biosciences, San Diego, CA). A total of 104 cells were analysed for each immunofluorescence profile. Shaded area, control cells treated with FITC-Avidin; solid line, FITC–MAA-stained cells; dotted line sialidase-treated cells followed by FITC–MAA-staining (PDF 29 kb) [file 10719_2012_9374_MOESM4_ESM.pdf]

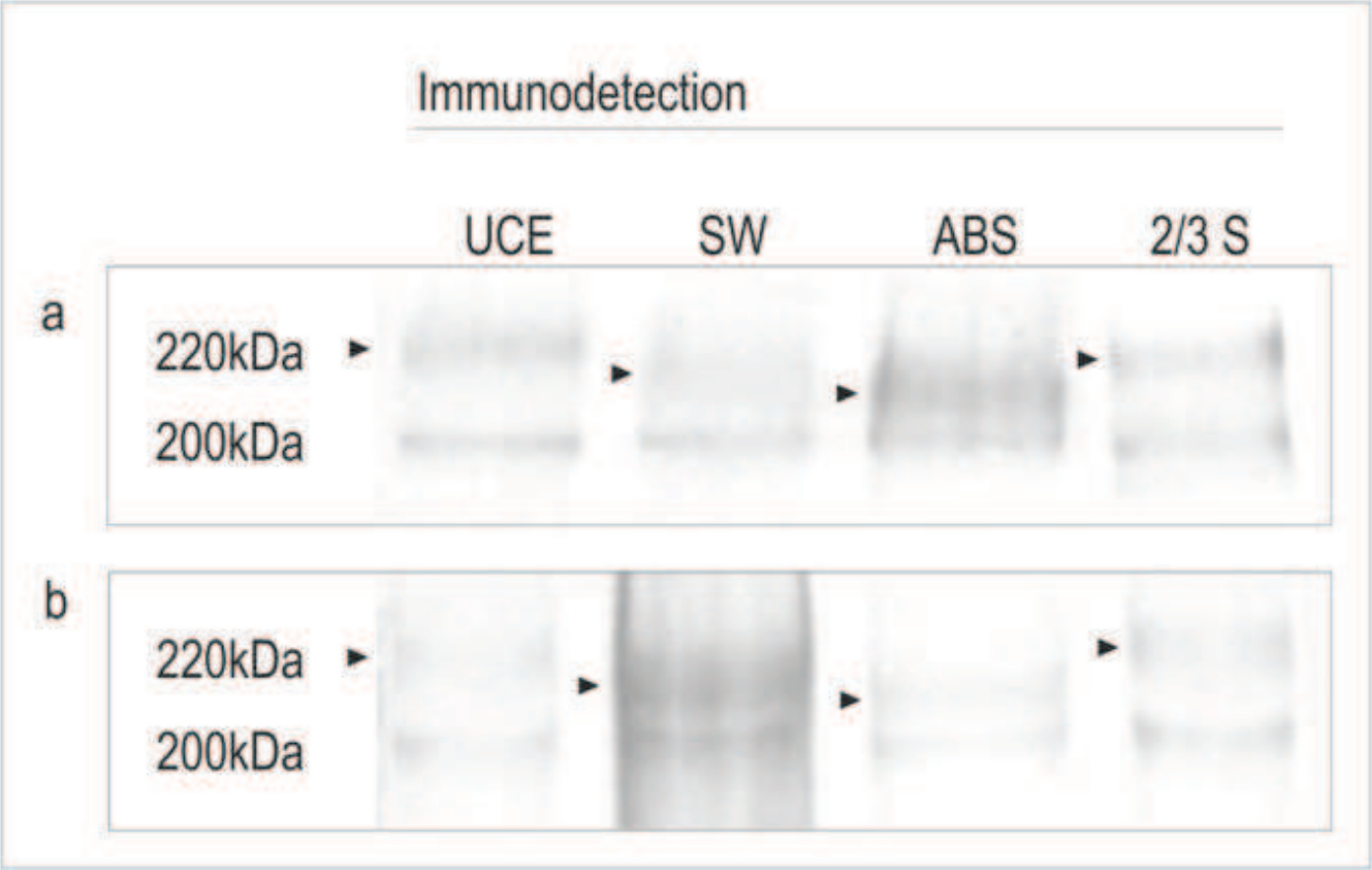

Supplement: Supplementary file 7 — Assessment of effectiveness of swainsonine and sialidase treatment of WM793 and WM1205Lu melanoma cells. (UCE) Untreated, (SW) swainsonine-treated, (ABS) A. ureafaciens sialidase-treated and (2/3S) S. pneumonia sialidase-treated cells were collected, protein cell extracts were prepared, and the effectiveness of treatment was assessed by western blotting. (PDF 44 kb) [file 10719_2012_9374_MOESM5_ESM.pdf]
